# Single dose moxidectin versus ivermectin for *Onchocerca volvulus* infection in Ghana, Liberia, and the Democratic Republic of the Congo: a randomised, controlled, double-blind phase 3 trial

**DOI:** 10.1016/S0140-6736(17)32844-1

**Published:** 2018-10-06

**Authors:** Nicholas O Opoku, Didier K Bakajika, Eric M Kanza, Hayford Howard, Germain L Mambandu, Amos Nyathirombo, Maurice M Nigo, Kambale Kasonia, Safari L Masembe, Mupenzi Mumbere, Kambale Kataliko, Jemmah P Larbelee, Mawolo Kpawor, Kpehe M Bolay, Fatorma Bolay, Sampson Asare, Simon K Attah, George Olipoh, Michel Vaillant, Christine M Halleux, Annette C Kuesel

**Affiliations:** a Department of Epidemiology and Biostatistics, School of Public Health, University of Health and Allied Sciences, Hohoe, Ghana; b WHO/AFRO, Brazzaville, Congo; c Centre de Recherche Clinique de Butembo (CRCB), Departement de la Recherche aux Cliniques du Graben, Université Catholique du Graben (UCG), Butembo, Democratic Republic of the Congo; d Kolahun Hospital, Kolahun, Liberia; e Ministère Provincial de la Santé, Kisangani, Democratic Republic of the Congo; f Department of Ophthalmology, Faculty of Medicine, Gulu University, Gulu, Uganda; g Nanomedicine Research Lab, CLINAM, University Hospital Basel, Basel, Switzerland; h Eye Care Services, Cooper Adventist Hospital, Monrovia, Liberia; i Liberia Institute for Biomedical Research (LIBR), Monrovia, Liberia; j Department of Chemistry and Biochemistry, South Dakota State University, Brookings, SD, USA; k Department of Microbiology, University of Ghana Medical School, Accra, Ghana; l Ghana Institute of Management and Public Administration, Centre for Management Development, Accra, Ghana; m Competence Center for Methodology and Statistics, Luxembourg Institute of Health, Strassen, Luxembourg; n UNICEF/UNDP/World Bank/WHO Special Programme for Research and Training in Tropical Diseases (TDR), WHO, Geneva, Switzerland

## Abstract

**Background:**

The morbidity and socioeconomic effects of onchocerciasis, a parasitic disease that is primarily endemic in sub-Saharan Africa, have motivated large morbidity and transmission control programmes. Annual community-directed ivermectin treatment has substantially reduced prevalence. Elimination requires intensified efforts, including more efficacious treatments. We compared parasitological efficacy and safety of moxidectin and ivermectin.

**Methods:**

This double-blind, parallel group, superiority trial was done in four sites in Ghana, Liberia, and the Democratic Republic of the Congo. We enrolled participants (aged ≥12 years) with at least 10 *Onchocerca volvulus* microfilariae per mg skin who were not co-infected with *Loa loa* or lymphatic filariasis microfilaraemic. Participants were randomly allocated, stratified by sex and level of infection, to receive a single oral dose of 8 mg moxidectin or 150 μg/kg ivermectin as overencapsulated oral tablets. The primary efficacy outcome was skin microfilariae density 12 months post treatment. We used a mixed-effects model to test the hypothesis that the primary efficacy outcome in the moxidectin group was 50% or less than that in the ivermectin group. The primary efficacy analysis population were all participants who received the study drug and completed 12-month follow-up (modified intention to treat). This study is registered with ClinicalTrials.gov, number NCT00790998.

**Findings:**

Between April 22, 2009, and Jan 23, 2011, we enrolled and allocated 998 participants to moxidectin and 501 participants to ivermectin. 978 received moxidectin and 494 ivermectin, of which 947 and 480 were included in primary efficacy outcome analyses. At 12 months, skin microfilarial density (microfilariae per mg of skin) was lower in the moxidectin group (adjusted geometric mean 0·6 [95% CI 0·3–1·0]) than in the ivermectin group (4·5 [3·5–5·9]; difference 3·9 [3·2–4·9], p<0·0001; treatment difference 86%). Mazzotti (ie, efficacy-related) reactions occurred in 967 (99%) of 978 moxidectin-treated participants and in 478 (97%) of 494 ivermectin-treated participants, including ocular reactions (moxidectin 113 [12%] participants and ivermectin 47 [10%] participants), laboratory reactions (788 [81%] and 415 [84%]), and clinical reactions (944 [97%] and 446 [90%]). No serious adverse events were considered to be related to treatment.

**Interpretation:**

Skin microfilarial loads (ie, parasite transmission reservoir) are lower after moxidectin treatment than after ivermectin treatment. Moxidectin would therefore be expected to reduce parasite transmission between treatment rounds more than ivermectin could, thus accelerating progress towards elimination.

**Funding:**

UNICEF/UNDP/World Bank/WHO Special Programme for Research and Training in Tropical Diseases.

## Introduction

Onchocerciasis, which is an infectious disease caused by the helminth *Onchocerca volvulus*, affects some of the world's most disadvantaged communities, 99% of which are in remote, rural areas in sub-Saharan Africa*.* Infective larvae, transmitted by the bite of *Simulium* spp, develop into macrofilariae that reside in subcutaneous and deep tissue nodules, and produce millions of microfilariae during their reproductive lifespan (9–11 years). Microfilariae live for 1–2 years primarily in the skin, from where they are taken up by the vectors, thus fuelling transmission. The host inflammatory reactions to dead microfilariae cause the dermatological, lymphatic, and ocular symptoms of onchocerciasis, including blindness due to microfilariae invading the eyes. The morbidity and socioeconomic effects of onchocerciasis have motivated large control programmes based now on community-directed treatment with ivermectin (CDTI) donated by Merck & Co (NJ, USA).^1^ At a dose of 150 μg/kg, ivermectin's so-called microfilaricidal effect substantially reduces skin and ocular microfilarial densities within days to weeks. Ivermectin's so-called embryostatic effect temporarily inhibits release of new microfilariae from the macrofilariae, resulting in skin microfilarial densities 1 year post treatment that are, on average, less than 40% of pretreatment values. This decrease reduces morbidity and parasite transmission.^1^, ^2^

**Research in context**

**Evidence before this study**

Evaluation of moxidectin for onchocerciasis control and elimination started with non-clinical pharmacology studies funded by TDR. At that time, elimination of onchocerciasis through mass administration of ivermectin was considered feasible in the small American foci, but not across Africa, which is where 99% of the at-risk population live. After external review of the non-clinical pharmacology and safety data, clinical evaluation of moxidectin began. The protocol for this study was based on one published and one unpublished healthy volunteer pharmacokinetic study and blinded safety data from the ongoing phase 2 study in people infected with *Onchocerca volvulus* in Ghana (now complete). No adverse events had occurred that would preclude administration in a larger study.

**Added value of this study**

This study confirmed the conclusions from the phase 2 study and five studies in healthy volunteers. Skin microfilarial densities were significantly lower after moxidectin than after ivermectin; the percentage of participants with undetectable microfilariae in skin was significantly higher after moxidectin than after ivermectin treatment to 18 months post treatment (final follow-up); and the safety profiles suggest moxidectin is suitable for mass treatment. Responses to ivermectin classified in areas with long-term ivermectin mass treatment as ‘suboptimal’ occurred in each of the four study areas without previous community-directed ivermectin treatment.

**Implications of all the available evidence**

Modelling and field studies done since the start of this study suggest that annual mass administration of ivermectin could eliminate onchocerciasis in many African foci, while other areas need alternative strategies, including more efficacious drugs. Furthermore, there are concerns about diminishing susceptibility of *O volvulus* to ivermectin's embryostatic effect in areas of long-term use. The suboptimal responses to ivermectin we observed show that data on changes in the frequency of such responses with duration of community-directed treatment with ivermectin are needed for conclusions about reduced *O volvulus* susceptibility or emerging resistance to ivermectin. Our data suggest that moxidectin could accelerate progress towards the elimination of onchocerciasis in Africa, including in areas with suboptimal responses to ivermectin.

In 11 of 13 foci in South and Central America (population about 0·56 million), 17–25 twice yearly mass administrations of ivermectin (with several years of quarterly treatments in hyperendemic communities) have or are likely to have eliminated onchocerciasis.^3^, ^4^ In 11 African countries, vector control, complemented later by mass administration of ivermectin, eliminated onchocerciasis as a public health problem. In the 20 other African countries where onchocerciasis is endemic, elimination as a public health problem has been achieved in regions where annual CDTI was implemented with high participation of the population for many years.^1^ In some areas (population about 25·4 million), elimination of transmission might have been achieved or is achievable within a few years.^5^ Where feasible, the target is to eliminate onchocerciasis in Africa by 2025.^6^

Questions remain about whether annual CDTI can eliminate onchocerciasis in areas with particularly high barriers to elimination, such as very high endemicity, loiasis co-endemicity that limits CDTI because of severe reactions in people with very high *Loa loa* microfilaraemia, and so-called suboptimal response to ivermectin. The WHO African Programme for Onchocerciasis Control (1995–2015) emphasised the need for alternative treatment strategies, including more efficacious drugs.^6^, ^7^

Moxidectin is a milbemycin macrocyclic lactone that is not registered for human use. Like ivermectin (an avermectin macrocyclic lactone), moxidectin is widely used by veterinarians.^8^ Moxidectin is minimally metabolised, has low affinity to p-glycoprotein transporters,^8^ and has a plasma half-life of 20–43 days in human beings,^9^, ^10^, ^11^, ^12^, ^13^ compared with less than 1 day for ivermectin.^8^ In a phase 2 study^14^ in people infected with *O volvulus*, moxidectin reduced and maintained low skin microfilarial density (SmfD; microfilariae per mg skin) in more participants, faster, to lower levels, and for much longer than did ivermectin. Our phase 3 study was designed to determine whether, 1 year after one moxidectin dose, SmfD was 50% or less than the SmfD was after one dose of ivermectin (ie, a superiority margin of ≥50%), and to collect additional safety data.

## Methods

### Study design and participants

Between April, 2009, and May, 2012, we did a randomised, double-blind, single oral dose, ivermectin-controlled study of moxidectin for superiority in onchocerciasis endemic areas in Ghana (Nkwanta district), Liberia (Lofa county), and the Democratic Republic of the Congo (Nord Ituri and Nord Kivu) without loiasis or previous CDTI.

We enrolled male and female volunteers aged 12 years or older, weighing 30 kg or more, who had 10 or more microfilariae per mg of skin. Participants with loiasis or lymphatic filariasis with an intensity of infection greater than 100 microfilariae per mL were to be excluded. Full eligibility criteria are shown in the appendix.

Volunteers gave consent or assent (with parental consent) through signature or thumbprint in the presence of a literate witness. SmfD was measured in the villages or nearby health clinics by study staff. Volunteers who qualified for further screening were brought to one of the research centres (Hohoe, Ghana; Bolahun, Liberia; Rethy, Nord Ituri and Butembo, Nord Kivu, Democratic Republic of the Congo) for 3 days of screening. Those who were eligible stayed another 5 days for treatment and initial follow-up, and were brought to the centre for 1–2 days for each follow-up visit.

This study was approved by the Ghana Food and Drugs Authority; Ghana Health Service Ethics Review Committee; Liberia Ministry of Health and Social Welfare; Ethics Committee of the Liberia Institute for Biomedical Research; Ministère de la Santé Publique of the Democratic Republic of the Congo; Ethics Committee of the Ecole de la Santé Publique Université de Kinshasa Democratic Republic of the Congo; and the WHO Ethics Review Committee. Study compliance with International Council for Harmonisation of Technical Requirements for Pharmaceuticals for Human Use Good Clinical Practice guidelines was monitored.^7^

### Randomisation and masking

At each centre, a pharmacist randomised participants using sponsor-provided computer-generated randomisation lists (block size 6) in a 2:1 ratio to 8 mg moxidectin or 150 μg/kg ivermectin, stratified by sex and level of infection (<20 *vs* ≥20 microfilariae per mg skin). Wyeth Research (NJ, USA) manufactured and provided 2 mg moxidectin tablets, purchased 3 mg ivermectin tablets from Merck & Co, overencapsulated both tablet types, and provided matching placebo. For each participant, the pharmacists prepared a sealed envelope, labelled with participant identifiers, that contained either four moxidectin-containing capsules or two, three, or four ivermectin-containing capsules plus two, one, or zero placebo capsules (as required by participant weight).

### Procedures

Each participant took the four capsules under observation. Clinical, ophthalmological, and laboratory examinations were done pretreatment, during the first 5 days, and 0·5, 1, 3, 6, 12, and 18 months post treatment (appendix). Given that the primary efficacy outcome was assessed at 12 months (chosen because moxidectin would likely be used annually), a protocol amendment removed follow-up at 18 months because of resource limitations after TDR became the sole sponsor in July, 2011. This change affected 256 participants (ie, those with 18-month follow-up due after ethics committee approval of the protocol amendment).

To quantify SmfD, we took four skin snips (one from each iliac crest and calf) at screening and at months 1, 6, 12, and 18 using a Holth corneoscleral punch. We weighed each snip before incubation for 8 h or more in isotonic saline. We then counted the microfilariae using a microscope and calculated SmfD as the arithmetic mean of microfilariae per mg skin across all snips.

An ophthalmologist did ocular examinations (visual acuity, visual fields, colour vision, intraocular pressure, fundus examination, slit lamp examination of anterior segment, and counting of live microfilariae in anterior chambers, living and dead microfilariae in cornea, and punctate opacities) before treatment, on day 3 or 4 after treatment, and at months 1, 6, 12, and 18.

We did physical examinations and vital sign measurements before treatment and at each follow up visit, and did serum biochemical, haematological, and urine analyses before treatment, on day 5 post treatment, and at months 0·5, 1, 3, and 6. We did an immunochromatographic card test for lymphatic filariasis during screening and tested for microfilaraemia in those who were positive. Since none of the participants were microfilaraemic, post-treatment tests were not conducted. We did one single-sample Kato-Katz test for intestinal helminths before treatment and, in those who were positive, at month 1. Details on testing for *Loa loa* infection in the sites in the Democratic Republic of the Congo and requirements for *Loa loa* testing in Liberia where potential participants might have lived in a loiasis endemic area during the preceding civil conflicts are provided in the appendix.

### Outcomes

The primary efficacy endpoint was SmfD at 12 months. Secondary efficacy measures were SmfD at months 1, 6, and 18, and the number of live microfilariae in anterior chambers at months 1, 6, 12, and 18 in participants with more than 10 live microfilariae in the anterior chambers across both eyes before treatment. Infection with intestinal helminths at month 1 was an exploratory outcome.

We assessed safety in terms of incidence of adverse events, including post-treatment changes in vital signs, symptoms, or laboratory values found through examinations, questions to participants, or spontaneous reporting. International Council for Harmonisation of Technical Requirements for Pharmaceuticals for Human Use criteria^7^ were used to determine whether adverse events were serious. We used the Onchocerciasis Chemotherapy Research Centre criteria (OCRC; appendix) to grade the severity of adverse events; these criteria were developed to grade symptoms of host inflammatory reactions to dead microfilariae. Such reactions present as symptoms of *O volvulus* infection and as adverse events after treatment with microfilaricidal drugs because of accelerated microfilariae death (ie, Mazzotti reactions).^14^, ^15^, ^16^

OCRC criteria for Mazzotti reactions differ substantially from National Cancer Institute criteria for grading similar events; OCRC criteria generally reflect much less severe symptoms grade for grade. National Cancer Institute criteria include need for medical intervention for grade 2 sometimes, grade 3 frequently, and grade 4 nearly always, whereas most OCRC criteria grade 4 Mazzotti reactions require no intervention. For symptoms not included in OCRC criteria, we used National Cancer Institute criteria (version 2.0) or provided our own method-specific criteria (appendix). Adverse events were characterised as Mazzotti reactions, non-Mazzotti adverse drug reactions, or adverse events not related to drugs on the basis of the temporal relationship to drug administration, participant general health, and known reactions to microfilaricidal or concomitant drugs. The characterisation of adverse events presented here is based on blinded, central review of all such data by the first author.^14^, ^17^, ^18^

### Statistical analysis

SmfD post-ivermectin depends on pretreatment SmfD determined by factors unknown during study planning (eg, local endemicity, control history, and participant lifetime risk of infection and treatment history). Therefore, we used post-ivermectin SmfD at month 12 from two previous studies (32·49 [SD 2·35] microfilariae per snip; 4·01 [2·41] microfilariae per mg) to calculate sample sizes to detect a difference of 50% or more in SmfD at month 12 with a two-sided Student's *t*-test (α=0·05 and 90% power with log_e_ [SmfD + 1] transformation), assuming SmfD post moxidectin is 0·5 SmfD post ivermectin, and 2:1 moxidectin:ivermectin randomisation (appendix). Even adjusting the larger resulting sample size (184:92) for 35% loss to follow-up was judged to give insufficient safety data. In consideration of the need for safety data, we planned for about 1000 participants in the moxidectin group and 500 in the ivermectin group to ensure that there was a high probability (moxidectin 0·99, ivermectin 0·92) for the detection of at least one adverse event with a true incidence of five in 1000,^14^ on the basis of the assumption that incidence follows a Poisson distribution.

We log transformed (y=log_e_ [SmfD + 1]) SmfD before analysis. In all mixed-effects models, the site was the random effect. The percentage treatment difference was calculated as the difference in adjusted geometric means (GM) as a percentage of post-ivermectin adjusted GM.

For the primary efficacy endpoint of SmfD at 12 months, we used a mixed-effects model for comparisons. Baseline SmfD, treatment, sex, level of infection, treatment × sex, and treatment × level of infection interactions were fixed effects. Sensitivity analyses included a non-parametric model (included in the protocol) and, following peer review, a fixed-effects model and a linear mixed-effects model of percentage change from baseline (appendix).

For the longitudinal analysis of SmfD at months 1, 6, 12, and 18, we used a mixed-effects model for repeated measures with participant as the statistical unit, baseline SmfD, treatment, sex, level of infection, time, treatment × sex, treatment × level of infection, and treatment × time interaction as fixed effects, and time as a repeated effect for the four SmfD measurements. We compared the number of participants with undetectable SmfD by use of a mixed-effects logistic model with treatment, sex, and level of infection as fixed effects. We calculated adjusted odds ratios. We used a mixed-effects model to compare the percentage reduction from baseline of live microfilariae in the anterior chambers of the eyes at month 12 with sex, level of infection, treatment, treatment × sex, and treatment × level of infection as fixed effects.

We used the Medical Dictionary for Regulatory Agencies (version 13.1) to code unrelated adverse events and adverse drug reactions. We used a specific dictionary (appendix) to code Mazzotti reactions because the Medical Dictionary for Regulatory Agencies distributes the same Mazzotti reactions (eg, pruritus) over several system organ classes or preferred terms, which compromises comparisons. To compare the overall number of participants with unrelated adverse events, adverse drug reactions, or Mazzotti reactions during the first 6 months post treatment, we used a two-tailed χ^2^ test. We compared by system organ classes and preferred terms or by Mazzotti reactions cluster, group, and sign or symptom, maximum grade, and seriousness across all ages and by age group (adolescents [12–17 years] *vs* adults [≥18 years]). Since the frequency and severity of Mazzotti reactions can depend on pretreatment SmfD,^19^ we also analysed these reactions by pretreatment SmfD (<20, 20 to <50, 50 to <80, and ≥80 microfilariae per mg). p values of 0·01 or lower were regarded as indicative of potential treatment differences, despite thousands of comparisons.

The primary population analysed was the modified intention-to-treat population (all participants who received study drug). Descriptive statistics included only those with applicable data. Analysis of reductions in live microfilariae in anterior chambers included only those with more than 10 live microfilariae in the anterior chambers across both eyes before treatment who had both eyes evaluated at month 12 (moxidectin group 131, ivermectin group 74).

We used SAS version 9.3 (SAS Institute, Cary, NC, USA) for statistical analyses. A data monitoring committee reviewed safety data during recruitment, including serious adverse events. This study is registered with ClinicalTrials.gov, number NCT00790998.

### Role of the funding source

The UNICEF/UNDP/World Bank/WHO Special Programme for Research and Training in Tropical Diseases (TDR) funded this study. TDR staff participated in study design, data management, analysis plan, interpretation, and manuscript writing. The corresponding author had full access to all data and made the final decision to submit for publication.

Because TDR was the sole sponsor of this study from July, 2011, resource limitations delayed availability of all results and dissemination at meetings to 2013–14. In 2014, the non-profit organisation Medicines Development for Global Health assumed sponsorship. To ensure publication and regulatory submissions using the same database, publication preparation was delayed until after Medicines Development for Global Health had completed its blinded database review in 2016. The manuscript was finalised without Medicines Development for Global Health involvement.

## Results

**Figure 1:**
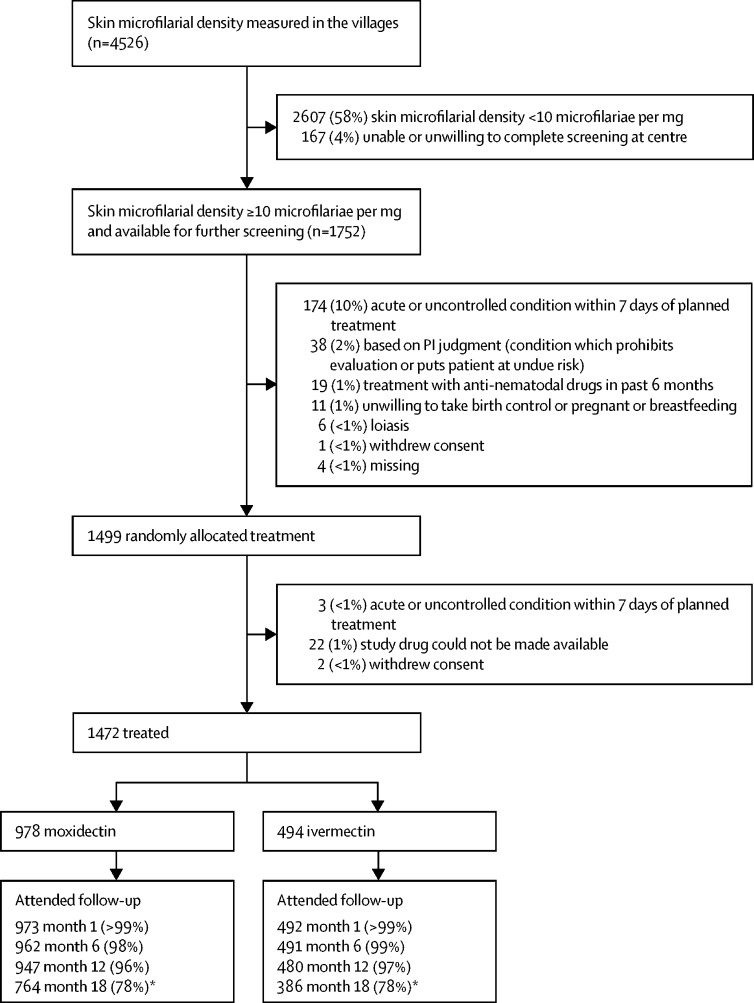
Trial profile *Low follow-up rate because the protocol was changed to remove 18-month visit.

We recruited participants between April 22, 2009, and Jan 23, 2011. Of 4526 people screened, 1472 (33%) were treated (figure 1). Participants' demographic characteristics and pretreatment characteristics related to *O volvulus* are shown in table 1. We have included other pretreatment characteristics, descriptive statistics and analysis outputs for all efficacy endpoints, including sensitivity analyses, results for intestinal helminths, and detailed safety data analyses in the appendix.

**Table 1:** Demographics and pre treatment characteristics of all participants treated

		**Moxidectin**	**Ivermectin**
Total participants		978	494
Participants in Nord Kivu, DR Congo		305	155
Participants in Nord Ituri, DR Congo		315	157
Participants in Lofa, Liberia		200	99
Participants in Nkwanta, Ghana		158	83
African origin		978 (100%)	494 (100%)
Age (years)		41·5 (16·4)	42·8 (16·1)
Adolescents (12–17 years old)		55 (6%)	24 (5%)
Weight (kg)		51·6 (8·40)	51·6 (7·90)
Height (cm)		158·9 (8·74)	159·4 (8·53)
Sex*			
	Males	626 (64%)	315 (64%)
	Females	352 (36%)	179 (36%)
Participants with <20 microfilariae per mg of skin*		281 (29%)	150 (30%)
Skin microfilariae density across all participants		38·8 (30·5)	41·2 (31·3)
Skin microfilariae density among ≥18 year-olds		39·4 (31·0)	41·9 (31·7)
Skin microfilariae density among 12–17-year-olds		29·2 (17·7)	27·0 (15·3)
Participants with >10 microfilariae in the anterior chamber across both eyes		136 (14%)	76 (15%)
Microfilariae in the anterior chamber across both eyes in participants with >10 microfilariae in the anterior chamber across both eyes		26·1 (19·6)	26·1 (18·2)

Data are arithmetic mean (SD) or n (%).

* Stratification variable.

Month 12 SmfD was lower in the moxidectin group (adjusted GM 0·6 microfilariae per mg [95% CI 0·3–1·0]) than in the ivermectin group (4·5 microfilariae per mg [3·5–5·9]; adjusted GM difference 3·9 microfilariae per mg [3·2–4·9], p<0·0001; treatment difference 86%). The difference in SmfD at month 12 was independent of sex (men 82%, women 91%), but differed between pretreatment level of infection (76% for <20 microfilariae per mg, 93% for ≥20 microfilariae per mg; p<0·0001; appendix).

**Figure 2:**
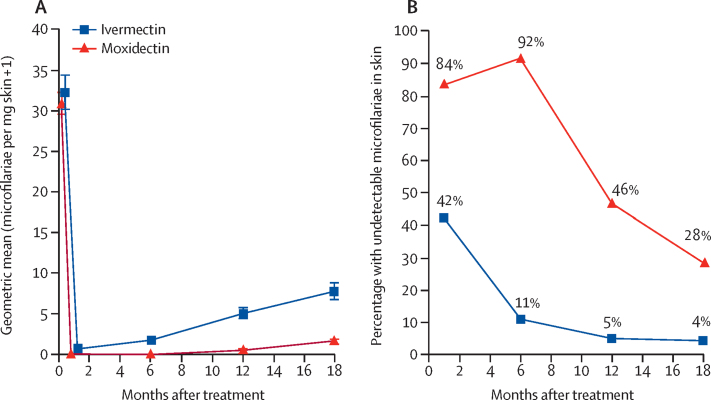
(A) Geometric mean (95% CI) of skin microfilarial density for all participants treated and (B) percentage of participants with undetectable skin microfilariae among all participants treated

SmfD was also lower at month 1 (by 86%), month 6 (by 97%), and month 18 (by 76%) after treatment with moxidectin than with ivermectin (figure 2A).

The proportion of participants with undetectable SmfD was higher in the moxidectin group than in the ivermectin group (p<0·0001; figure 2B; appendix). The number of participants with undetectable SmfD from month 1 to month 12 was 360 (38%) of 938 in the moxidectin group and seven (2%) of 478 in the ivermectin group.

**Figure 3:**
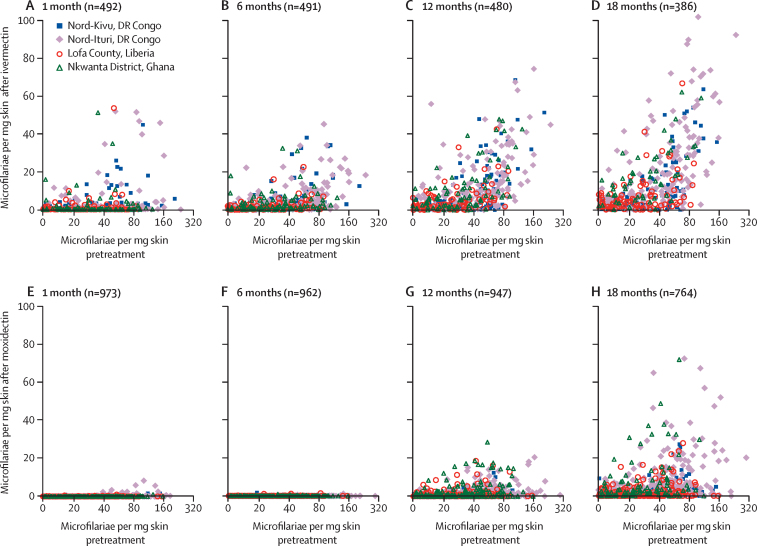
Skin microfilarial density at 1, 6, 12, and 18 months after treatment with ivermectin (A–D) and after treatment with moxidectin (E–G) versus pretreatment X-axis shows pretreatment skin microfilarial density on a logarithmic scale; y-axis shows post-treatment skin microfilarial density on an arithmetic scale.

In each study area, the spread of post-treatment SmfD was greater among people treated with ivermectin compared with moxidectin (figure 3). For ivermectin, minimum SmfD from month 1 to month 12 was 1% or less of pretreatment SmfD in 64% of people and more than 10% of pretreatment SmfD in 11% of people. Maximum SmfD from month 1 to month 12 was 1% or less of pretreatment SmfD in 4% of people, more than 20% in 44% of people, and more than 40% in 20% of people. For moxidectin, minimum SmfD from month 1 to month 12 was 1% or less of pretreatment SmfD in 100% of people. Maximum SmfD from month 1 to month 12 was 1% or less of pretreatment SmfD in 58%, more than 20% in 3% of people, and more than 40% in 1% of people (appendix).

In participants with more than 10 live microfilariae across the anterior chambers of both eyes before treatment, the number of these microfilariae decreased slowly from pretreatment to month 6. At month 12 in the moxidectin group, the number of participants without detectable microfilariae was 126 (96%) of 131; at month 18, this value was 113 (98%) of 115. In the ivermectin group, 62 (84%) of 74 participants did not have detectable microfilariae at month 12; this value was 55 (90%) of 61 at month 18. The percentage reduction in live microfilariae in the anterior chambers from pretreatment to month 12 did not differ between the two treatments (p=0·13; appendix).

Both drugs were well tolerated. Table 2 shows participants with at least one adverse event during the 6 months following treatment. None of the serious adverse events were related to the drugs given. In each treatment group, two participants died within 6 months of the treatment for reasons unrelated to the study (appendix).

**Table 2:** Participants with adverse events during the first 6 months after treatment by adverse event category and severity (grade)

				**All participants**		**Adolescents (12–17 years)**	
				Moxidectin	Ivermectin	Moxidectin	Ivermectin
Number of participants				978	494	55	24
Serious adverse events							
	Any grade			39 (4%)	17 (3%)	0	0
	1			1 (<1%)	0	0	0
	2			12 (1%)	9 (2%)	0	0
	3			21 (2%)	4 (1%)	0	0
	4			12 (1%)	5 (1%)	0	0
Unrelated adverse events							
	Any grade			950 (97%)	483 (98%)	50 (91%)	23 (96%)
	1			897 (92%)	458 (93%)	48 (87%)	23 (96%)
	2			611 (63%)	305 (62%)	13 (24%)	1 (4%)
	3			202 (21%)	115 (23%)	6 (11%)	1 (4%)
	4			63 (6%)	36 (7%)	1 (2%)	1 (4%)
Non-Mazzotti adverse drug reactions							
	Any grade			0	0	0	0
Mazzotti reactions							
	Any grade			967 (99%)	478 (97%)	55 (100%)	22 (92%)
	1			918 (94%)	430 (87%)	55 (100%)	20 (83%)
	2			711 (73%)	339 (69%)	35 (64%)	8 (33%)
	3			303 (31%)	145 (29%)	12 (22%)	3 (13%)
	4			321 (33%)	178 (36%)	20 (36%)	11 (46%)
		Ocular Mazzotti reactions					
			Any grade	113 (12%)	47 (10%)	8 (15%)	3 (13%)
			1	101 (10%)	39 (8%)	8 (15%)	3 (13%)
			2	15 (2%)	7 (1%)	0	0
			3	3 (<1%)	2 (<1%)	0	0
			4	0	0	0	0
		Laboratory Mazzotti reactions*					
			Any grade	788 (81%)	415 (84%)	43 (78%)	16 (67%)
			1	373 (38%)	193 (39%)	14 (26%)	4 (17%)
			2	346 (35%)	178 (36%)	16 (29%)	5 (21%)
			3	196 (20%)	91 (18%)	11 (20%)	2 (8%)
			4	266 (27%)	163 (33%)	15 (27%)	9 (38%)
		Clinical Mazzotti reactions					
			Any grade	944 (97%)	446 (90%)	54 (98%)	18 (75%)
			1	859 (88%)	398 (81%)	51 (93%)	17 (71%)
			2	567 (58%)	253 (51%)	29 (53%)	4 (17%)
			3	136 (14%)	61 (12%)	1 (2%)	1 (4%)
			4	90 (9%)†	25 (5%)†	7 (13%)	2 (8%)

Data are n (%). The numbers of participants at a given grade within each category or subcategory of events do not sum up to the total number for that category or subcategory because some participants had different types of events with different grades within the same category or subcategory. For participants with more than one episode of the same type of event at different levels of severity, we recorded the most severe grade. Adverse events were classified as serious or non-serious and as non-Mazzotti adverse drug reactions (ie, treatment-related), Mazzotti reactions (ie, adverse events related to accelerated microfilarial death after treatment with microfilarial drugs), or unrelated adverse events as explained in the Methods.

* Changes in laboratory values considered Mazzotti reactions are most frequently haematological (eosinopenia followed by eosinophilia, lymphocyte decrease followed by lymphocytosis) but might also affect serum biochemistry (most frequently aspartate and alanine aminotransferase; appendix).

† p=0·010.

The profiles of Mazzotti reactions after both treatments were similar when analysed by severity across all pretreatment SmfD (table 2) and by pretreatment SmfD to take into account that such reactions reflect host response to the microfilaricidal effect and their frequency and severity can increase with pretreatment SmfD (appendix).^19^ Grade 4 clinical Mazzotti reactions (OCRC criteria) were more frequent among people treated with moxidectin than with ivermectin because severe symptomatic postural hypotension was more common in the moxidectin group (45 [5%] of 978 *vs* seven [1%] of 494). Severe symptomatic postural hypotension is diagnosed when a person, after 5 or more min in the supine position, is unable to stand up and be still for 2 min because of dizziness or weakness linked to a decrease in blood pressure. Such hypotension does not require intervention and resolves quickly after the individual lies down. People in the moxidectin group had severe symptomatic postural hypotension from treatment day to 2 days post treatment, compared with from 1–3 days post treatment in those treated with ivermectin.

The most frequent ocular Mazzotti reactions were pruritus (moxidectin 43 [4%] of 978 participants, ivermectin 12 [2%] of 494 participants), conjunctivitis (moxidectin 40 [4%] participants, ivermectin 15 [3%] participants), and eye pain (moxidectin 29 [3%] participants, ivermectin 8 [2%] participants). Fewer than 1% of participants had eyelid swelling, ocular discomfort, tearing or watery eyes, blurred vision, and photophobia. Ocular Mazzotti reactions exceeding grade 1 occurred in 1% or fewer participants in both treatment groups, the most frequent being eye pruritus (1%).

## Discussion

Moxidectin was well tolerated without substantial adverse drug reactions, confirming data from the previous study in people infected with *O volvulus*^14^ and five studies in healthy volunteers.^9^, ^10^, ^11^, ^12^, ^13^ Severe symptomatic postural hypotension—which resolves quickly without treatment—was the only efficacy-related OCRC criteria grade 4 reaction that was more common in the moxidectin than in the ivermectin group. Such hypotension was reported in up to 22% of ivermectin-treated participants in clinical studies,^17^ but rarely in large-scale ivermectin treatment.^19^ Our data suggest that moxidectin is as compatible with mass drug administration as ivermectin is, which was given to more than 110 million people in 2014.^20^

The significantly lower SmfD (ie, parasite transmission reservoir) from month 1 to month 18 after moxidectin than after ivermectin—with 86% treatment difference at month 12—confirms our efficacy-related study hypothesis and is consistent with previous data.^14^ Reduced SmfD would lead to decreased parasite transmission and thus shorter time to onchocerciasis elimination with annual mass administration of moxidectin compared with ivermectin. Modelling of the phase 2 study data^14^ estimated the number of years to preliminary operational thresholds for interrupting treatment (as defined by the African Programme for Onchocerciasis Control) to be 30–40% lower with annual mass administration of moxidectin than with ivermectin and comparable with those modelled for twice yearly mass administration of ivermectin.^21^

Moxidectin would be particularly useful to accelerate the elimination of onchocerciasis in situations where there are operational barriers to mass drug administration (such as community accessibility, conflict or civil war, or health services support for communities), where precontrol endemicity was so high that infection prevalence is still high despite long-term CDTI, and where transmission seasons are long or have two peaks.^21^ Interventions can only be discontinued when the whole transmission zone (an area sharing a parasite population) meets the discontinuation criteria. Therefore, moxidectin could also be useful in situations where one area within a transmission zone needs to accelerate progress towards elimination to meet CDTI discontinuation criteria at the same time as other areas that have benefitted from earlier or better implemented CDTI (achieved, for example, through better advocacy, community ownership, self-monitoring and participation, or fewer systematic non-compliers^6^) or had lower pretreatment endemicity. In areas where onchocerciasis and loiasis are coendemic, moxidectin could advance the elimination of onchocerciasis within a test-not-treat strategy that excludes people with *Loa loa* microfilaraemia that puts them at risk of serious adverse events from microfilaricidal drugs.^22^ A safety-focused study needs to be done to establish the appropriate risk threshold.

Persistent onchocerciasis prevalence and suboptimal responses to ivermectin's embryostatic effect after long-term CDTI in Ghana and Cameroon have raised concerns about decreasing *O volvulus* susceptibility to ivermectin.^23^, ^24^, ^25^, ^26^, ^27^ Suboptimal responses are characterised by an earlier and higher SmfD increase than is considered normal based on prior experience or meta-analysis (appendix); such responses are seen even during biannual CDTI.^2^, ^23^, ^24^, ^25^, ^26^, ^27^, ^28^ With suboptimal responder progeny in the skin earlier and for longer between CDTI rounds, such parasites could be preferentially transmitted, leading to an increase in their prevalence and a gradual decrease in the effect of CDTI on transmission. The high rate of optimal responses to moxidectin (undetectable SmfD at months 1, 6, and 12) suggests that moxidectin could accelerate progress towards elimination in areas with high prevalence of suboptimal responses to ivermectin.

In each of the four CDTI-naive study areas, some participants met the criteria for suboptimal response to ivermectin's embryostatic effect or for suboptimal response to ivermectin's microfilaricidal effect.^14^ This finding raises the question of whether suboptimal responses in Ghana and Cameroon are now more common than they were at CDTI initiation and suggests that data on the variability of interindividual responses and relative frequencies of different response levels are required to detect changes in *O volvulus* susceptibility to ivermectin over time.

The biological mechanism(s) through which moxidectin leads to lower long-term SmfD than does ivermectin are unknown.^14^ They probably include a combination of microfilaricidal, embryostatic, embryotoxic, and macrofilaricidal effects, or a reproductive life-span shortening effect.^2^, ^8^, ^14^ Our study did not include an evaluation of macrofilariae, which might have added new data. Moxidectin's superiority is probably related to its long half-life of 20–43 days,^9^, ^10^, ^11^, ^12^, ^13^ compared with ivermectin (<1 day).^8^

This study and the phase 2 study^14^ were single-dose studies. Given the sustained effect (6–12 months) of a single moxidectin dose on SmfD, administration of another dose of moxidectin before the effect of the previous dose has fully waned could have cumulative effects. A large study comparing multiple annual and biannual moxidectin and ivermectin treatments could provide sufficient macrofilariae from palpable nodules to identify cumulative effects on the reproductive capacity and viability of macrofilariae and the relative contribution of effects on microfilariae and macrofilariae on moxidectin's superior effect on SmfD;^14^ provide a better basis for modelling the relative benefits and costs of these regimens to reduce time to onchocerciasis elimination;^21^ provide a better estimate of the relative frequency of rare Mazzotti reactions; provide more data on the relative efficacy against intestinal helminths; and provide more data to inform community mobilisation strategies and materials for information, education, and communication.

Our study did not include a paediatric population. A pharmacokinetic study to determine a safe dose in children is required.

For countries to consider the use of moxidectin for the control of onchocerciasis, moxidectin needs to be registered and manufactured. In 2014, WHO licensed all moxidectin-related data at its disposal to Medicines Development for Global Health. The US Food and Drug Administration assigned the new drug application submitted by Medicines Development for Global Health to priority review. Medicines Development for Global Health is preparing a paediatric pharmacokinetic study and a large multi-dose comparative study^7^ and plans to evaluate moxidectin's benefit for lymphatic filariasis, strongyloidiasis, soil-transmitted helminthiasis, and scabies.^7^, ^29^
