# Department of Error

**DOI:** 10.1016/S0140-6736(18)32403-6

**Published:** 2018-10-06

*Opoku NO, Bakajika DK, Kanza EM, et al. Single dose moxidectin versus ivermectin for Onchocerca volvulus infection in Ghana, Liberia, and the Democratic Republic of the Congo: a randomised, controlled, double-blind phase 3 trial.* Lancet *2018; **392:** 1207–16—*In this Article (published online first on Jan 17, 2018), the number of people who received ivermectin in 2014 should have been more than 110 million, and George Olipoh and Asare Sampson should have been included as authors rather than listed in the Acknowledgments section. These corrections have been made to the online version as of Oct 4, 2018. The printed Article is correct.

