# Supplementary material for: Single dose moxidectin versus ivermectin for Onchocerca volvulus infection in Ghana, Liberia, and the Democratic Republic of the Congo: a randomised, controlled, double-blind phase 3 trial
Source: Lancet. 2018 Oct 6;392(10154):1207–16. doi: 10.1016/S0140-6736(17)32844-1 (PMC6172290; doi:10.1016/S0140-6736(17)32844-1)
Supplement: Supplementary appendix [file mmc1.pdf]

THE LANCET

Supplementary appendix

This appendix formed part of the original submission and has been peer reviewed. We post it as supplied by the authors.

Supplement to: Opoku NO, Bakajika DK, Kanza EM, et al. Single dose moxidectin versus ivermectin for *Onchocerca volvulus* infection in Ghana, Liberia, and the Democratic Republic of the Congo: a randomised, controlled, double-blind phase 3 trial. *Lancet* 2018; published online Jan 17. http://dx.doi.org/10.1016/S0140-6736(17)32844-1.

Supplemental Appendix

To: Opoku et al. Single dose moxidectin versus ivermectin for Onchocerca volvulus infection in Ghana, Liberia, and the Democratic Republic of the Congo: a randomised, controlled, double-blind phase 3 trial

Contents

1 Methods 3

1.1 Eligibility criteria 3

1.1.1 Inclusion Criteria 3

1.1.2 Exclusion Criteria 3

1.2 Overview of examinations conducted 3

Table S 1: Type and timing of examinations conducted 3

1.3 Onchocerciasis Chemotherapy Research Centre (OCRC) and other protocol specified adverse event grading criteria 4

Table S 2: Onchocerciasis Chemotherapy Research Centre Criteria (OCRC-C) 5

Table S 3: Grading of visual fields abnormalities measured via FDT perimetry 12

Table S 4: Grading of colour vision with PV16 12

Table S 5: Grading of laboratory events not included in other grading scales provided 12

1.4 Mazzotti reactions 12

1.4.1 Overview of Mazzotti reactions 12

1.4.2 Coding of adverse events characterized as Mazzotti reactions 13

Table S 6: Dictionary for coding signs and symptoms of onchocerciasis and AEs characterized as Mazzotti reactions into reaction clusters and groups 13

1.5 Sample size calculation 14

1.6 Statistical analysis 15

1.6.1 Statistical methods for primary efficacy endpoint 15

1.6.2 Analysis populations 15

1.7 Overview of protocol amendments 15

2 Results 16

2.1 Baseline characteristics 16

2.1.1 Skin microfilariae 16

Table S 7: Skin microfilariae density in subgroups defined by level of infection at baseline and presence of palpable nodules pre-treatment 16

Figure S 1 A: Number of nodules palpated pre-treatment vs pre-treatment skin microfilariae density by sex of participants; B: Participant age vs. number of palpable nodules pre-treatment. 16

Figure S 2 Skin microfilariae density pre-treatment by age and sex 17

2.1.2 Results of ICT testing pre-treatment 17

2.1.3 Intestinal helminth infection pre-treatment 17

Table S 8: Intestinal helminth infections at baseline by site 17

2.2 Efficacy data 18

2.2.1 Primary efficacy endpoint (SmfD 12 months post-treatment) analysis with protocol-specified mixed effects model 18

Table S 9: Primary efficacy endpoint: Descriptive statistics and protocol-specified mixed-effects model analysis 18

2.2.2 Protocol-planned non-parametric analysis of primary efficacy endpoint (SmfD 12 months post-treatment) 19

2.2.3 Sensitivity analysis of primary efficacy endpoint in response to statistical peer review: Fixed effects model 19

Table S 10: Primary efficacy endpoint: Fixed effects model sensitivity analysis output 19

2.2.4 Sensitivity analysis in response to statistical peer review: Descriptive statistics and linear mixed model of percentage SmfD change from baseline at month 12 19
Table S 11: Descriptive statistics and linear mixed model analysis output for percentage SmfD change from baseline at month 12 19
2.2.5 Secondary efficacy endpoints 20
Table S 12: Skin microfilariae density 1, 6, 12 and 18 months after treatment: Descriptive statistics and mixed effects model output 20
Table S 13: Percentage SmfD change from baseline of skin microfilariae density 1, 6, 12 and 18 months after treatment: Descriptive statistics 20
Table S 14: Proportion (%) of participants with undetectable levels of skin microfilariae 1, 6, 12 and 18 months after treatment: Descriptive statistics and adjusted odds ratios 21
Figure S 3 Percentage of participants with 1, 6, and 12 months follow up data treated with ivermectin (A, C, n=478) and moxidectin (B, D, n=938) by minimum (A, B) and maximum (C, D) skin microfilariae densities between 1 and 12 months in percent of pre-treatment skin microfilariae density 21
Table S 15: Microfilariae levels in the anterior chamber 12 months post treatment among participants with > 10 mf across both anterior chambers at baseline and Month 12 data for both eyes: Descriptive statistics 22
Table S 16: Reduction (%) from baseline of microfilariae levels in the anterior chamber 12 months post treatment among participants with > 10 mf across both anterior chambers at baseline and Month 12 data for both eyes): Descriptive statistics and model output 22
2.3 Safety data 22
2.3.1 Serious Adverse Events 22
Table S 17: Serious adverse events (SAEs) during the first 6 months post treatment (MedDRA coding) 22
2.3.2 Adverse events reported as resulting in Death (MedDRA coding) 23
Table S 18: Narratives generated from SAE reports for AEs with outcome death 23
2.3.3 Dependency of Mazzotti reactions on pre-treatment skin microfilariae density 25
Figure S 4 Percentage of participants overall and by skin microfilariae density pre-treatment with at least one MAZ of any grade and by highest grade experienced. (A) Any type of MAZ, (B) any type of clinical MAZ, (C) any type of ocular MAZ, (D) any type of haematological MAZ, (E) any type of biochemical MAZ. 25
Figure S 5 Percentage of participants overall and by intensity of infection pre-treatment with at least one clinical MAZ of any grade and by highest grade experienced for the specified clinical MAZ group. 26
Figure S 6 Percentage of participants overall and by intensity of infection pre-treatment with the specified hematological or serum biochemical MAZ by highest grade experienced 27
Figure S 7 Percentage of participants overall and by intensity of infection pre-treatment with the specified types of ocular MAZ of any grade and by highest grade experienced 28
2.4 Exploratory endpoint: Effect on intestinal helminths 29
Table S 19: Cure rate and egg reduction rate across all sites for hookworm, roundworm and whipworm 29
Figure S 8 Individual data based analysis of response of hookworm infection to moxidectin and ivermectin 29
3 Classification of Response to ivermectin in previous studies 29
Table S 20: Criteria in previous studies for classification of response to ivermectin treatment based on skin microfilariae levels 30
4 References 30

1 Methods

1.1 Eligibility criteria

Eligibility criteria were designed to include a population as close to a potential mass treatment population as possible without subjecting participants to higher than necessary unknown risks (exclusion of <12 years in absence of paediatric PK data, pregnant or breastfeeding women, *Loa loa* co-infection (study areas were not loiasis endemic)), compromising efficacy assessment (exclusion of people with anti-nematodal treatment in last 6 months and plans to move out of the area), confounding adverse event (AE) causality assessment (exclusion of people with acute or uncontrolled disease in the 7 days pre-treatment, treated with an investigational drug or device in the 4 weeks before study drug administration, with lymphatic filariasis (LF) microfilaremia > 100 mf/ml).

1.1.1 Inclusion Criteria

- Male or female subjects ≥ 12 years of age and weighing ≥ 30 kg.
- Subjects with *Onchocerca volvulus* infection, at least 10 microfilariae/mg by skin snip.
- All female subjects not surgically sterile or postmenopausal had to commit to the use of a reliable method of birth control for 6 months after study drug administration. (Birth control devices / drugs chosen by the woman - and partner, if applicable - was provided by the study)

1.1.2 Exclusion Criteria

- Treatment with the anti-nematodal drugs diethylcarbamazine [DEC], suramin, ivermectin, albendazole or levamisole, within 6 months before planned study drug administration.
- Pregnant or breastfeeding women.
- Low probability of residency in the area (based on subject's assessment) over the 20 months following the anticipated date of study drug administration.
- Subjects with loiasis.
- Subjects with lymphatic filariasis (LF) with an intensity of infection >100 mf/mL.
- Acute or uncontrolled disease process (e.g., acute pneumonia requiring therapy or end stage AIDS) within 7 days before study drug administration. Patients with stable chronic diseases (e.g., no change in medication for past month) were permitted.
- Any investigational drugs or investigational devices within 4 weeks before study drug administration that may confound safety and/or efficacy assessments.
- Known or suspected allergy to moxidectin or ivermectin or other compounds related to these classes of medication.
- Any concomitant condition that, in the opinion of the investigator, would preclude an evaluation of a response or would place subject's health at undue risk.

1.2 Overview of examinations conducted

Table S 1: Type and timing of examinations conducted

	Screening/ Baseline[9]	D1	D2-4	D6 (±1D)	D14 (±2D)	M1 (±5D)	M3 (±0.5M)	M6 (± 1M)	M12 (± 1M)	M18 (± 2M)
**Safety**										
Weight	X					X	X	X		
Physical examination[1]	X	X	X	X	X	X	X	X	X	X
Vital signs[2]	X	X	X	X	X	X	X	X	X	X
12 lead ECG	X		2 or 3							
Ocular Examination[3]	X		3 or 4			X		X	X	X
Laboratory examination[4]	X			X	X	X	X	X		
Loa loa screening [5]	X									
Questioning	X	X	X	X	X	X	X	X	X	X
**Efficacy**										
Skin snips[6]	X					X		X	X	X
Ocular examination[3]	X		3 or 4			X		X	X	X
**Co-infections**										
Kato-Katz[7]	X					X				
Lymphatic filariasis[8]	X								X	X

D: Day, M: Month. X indicates examination at each follow-up indicated in the top row, numbers indicate the day for performing the examination.

1 Physical examination including neurological examination. Subcutaneous nodule palpation was conducted at screening and Month 12 and 18.
2 Vital signs including temperature, respiratory rate, pulse rate (PR) and blood pressure (BP) after at least 5 minutes supine. Up to Month 1, PR and BP measurement were repeated after 2 minutes standing following ≥ 5 minutes supine at baseline. Day 1: once before and once after drug administration.
3 Ocular Examination included visual acuity (Snellen E), visual fields (FDT perimetry), colour vision (PV16 colour vision test), intraocular pressure, examination of the fundus, slit lamp examination of anterior segment, counting of microfilariae in anterior chamber, living and dead microfilariae in cornea and punctate opacities.
4 Laboratory evaluations included: serum biochemistry (Na^+, K^+, Cl^-, glucose, total protein, albumin, urea, creatinine, alkaline phosphatase, lactic dehydrogenase, total bilirubin, gamma-glutamyl transpeptidase, aspartate aminotransferase, and alanine aminotransferase), haematology (complete blood cell count, haematocrit, haemoglobin, 5-part differential white blood cell count, platelet count), dipstick semiquantitative urinalysis (specific gravity, pH, albumin (protein), glucose, ketones, haemoglobin, bilirubin, urobilinogen, nitrite and leukocyte esterase). Microscopic evaluation of the urine for red and white blood cells, epithelial cells, bacteria, casts, and crystals was performed at baseline and thereafter only at investigator's discretion as medically indicated.
5 Study sites were selected for lack of *Loa loa* endemicity. Testing for loiasis infection was conducted in the two sites in DRC since potential study participants might have lived in a loiasis endemic area during the preceding civil conflict. Blood was collected between approximately 11:00 and 14:00 by finger prick with a 60 µL non-heparinized capillary tube. The blood was spread on a slide, dried, Giemsa stained and dried at ambient temperature. *Loa loa* microfilariae were counted at a magnification of 100.
6 Minimum of 1 mg from each iliac crest and calf with a 2 mm corneoscleral Holth punch, incubated individually in physiological saline for at least 8 hours.
7 Single sample Kato Katz for intestinal helminths. Follow up sample obtained only in those infected pre-treatment.
8. Lymphatic filariasis (LF) evaluation in areas coendemic for LF or where endemicity is unknown. Pretreatment: immunochromatographic card test for *Wuchereria bancrofti* (ICT) in subjects without clinical signs and symptoms. Nightblood examination for microfilaria for ICT positive and all subjects with signs and symptoms of LF. Post-treatment LF evaluation via nightblood evaluation only in subjects positive at baseline; ICT at Month 12 in subjects with positive baseline ICT (with microfilaraemia testing if positive at baseline); ICT at Month 18 in subjects with positive Month 12 ICT (with microfilaraemia testing if ICT-positive at Month 18).
9 Skin snips for eligibility assessment were obtained in the village up to 60 days before study drug administration. All other examinations for eligibility assessment and determination of baseline status were done on days -3 to -1 in the study centre.

1.3 Onchocerciasis Chemotherapy Research Centre (OCRC) and other protocol specified adverse event grading criteria

Table S 2, Table S 3, Table S 4 and Table S 5 show the OCRC grading criteria as well as other protocol specified criteria for protocol-specified methods and laboratory values not included in the OCRC grading criteria or the NCI CTC v 2.0 criteria (http://ctep.cancer.gov/protocolDevelopment/electronic_applications/ctc.htm).

Table S 2: Onchocerciasis Chemotherapy Research Centre Criteria (OCRC-C)

Sign/Symptom	Grade 0	Grade 1	Grade 2	Grade 3	Grade 4
**MAZZOTTI REACTION CRITERIA - SYSTEMIC REACTIONS (1)**					
Itching	None	Mild	Moderate or 'Severe' but with only occasional scratching	'Severe' and with fairly vigorous scratching "Windmill Effect"	'Severe' and with restlessness, agitation, loss of epithelium or prolonged vigorous scratching
Note: 'Severe' is patient's perception of grade of discomfort. "Windmill Effect" = arms in fairly continuous motion with scratching					
Headache	None	Mild	Moderate or 'Severe' but patient comfortable	'Severe' and with obvious distress	Unbearable
Note: 'Severe' is patient's perception of grade of discomfort					
Joint pain (arthralgia)	None	Mild	Moderate or 'Severe' but without change in gait or function	'Severe' and with a definite limp or change in function due to joint pain	'Severe' and with marked interference with motion or function (commonly "Pillar of Salt" Effect)
Note: 'Severe' is patient's perception of grade of discomfort. "Pillar of salt Effect" = rooted to the spot and unable to walk due to severe pain					
Muscle pain (myalgia)	None	Mild	Moderate or 'Severe' but without change in gait or function	'Severe' and with a definite limp or change in function due to muscle pain	'Severe' and with marked interference with motion or function (commonly "Pillar of Salt" Effect)
Note: 'Severe' is patient's perception of grade of discomfort. "Pillar of salt Effect" = rooted to the spot and unable to walk due to severe pain					
Gland pain	None	Mild	Moderate or 'Severe' but without change in gait or function	'Severe' and with a definite limp or change in function due to gland pain ("Hydrocele Gait" may be present)	'Severe' and with marked interference with motion or function (commonly "Pillar of Salt" Effect; "Knee Elbow Position")
Note: 'Severe' is patient's perception of grade of discomfort. "Pillar of salt Effect" = rooted to the spot and unable to walk due to severe pain<br>"Hydrocele Gait" = Walking on a broad base with trunk slightly flexed.					
Gland tenderness	None	Very firm pressure needed to elicit pain	Moderate pressure elicits pain	Very light touch elicits severe pain	Patient refuses palpation on account of severe pain
Note: Assessment carried out by Medical Officer					
Rash	None	< ⅓ body surface	⅓-< ⅔ body surface	≥⅔ body surface	-
Note: Assessment carried out by Medical Officer					
**MAZZOTTI REACTION CRITERIA - SYSTEMIC REACTIONS (2)**					
Temperature increase	$<38^{0}$C	38.0 - 39.0°C	39.1 - 40.0°C	> 40.0°C for < 24hrs	> 40.0°C > 24hrs
Increase in Pulse Rate in beats per minute	0 -20	>20-<36	≥36-<52	≥52-<68	≥68
Note: Readings taken lying as well as after standing for 2 minutes					
Fall in Mean Arterial Pressure	0-20	>20-<25	≥25-<30	≥30-<35	≥35 or CNS*
Note: Readings are taken lying as well as after standing for 2 minutes. * CNS means Could Not Stand long enough for pressure to be taken due to severe postural hypotension					
Increase in Respiratory Rate per minute	0-<6	≥6-<12	≥12-<18	≥18-<24	≥24
Note: Contributes little to overall score					
**MAZZOTTI REACTION CRITERIA - OTHER REACTIONS**					
Lymphatics	Normal	Mild lymphedema	Moderate lymphedema requiring compression; lymphocyst	Severe lymphedema limiting function; lymphocyst requiring surgery	Severe lymphedema limiting function with ulceration
Note: An acute brawny edema of one or more limbs is a manifestation of the Mazzotti reaction					
Facial swelling	None	Mild swelling	Moderate swelling	Severe with eyes completely shut	-
Note: A fairly common manifestation of the Mazzotti reaction					

Sign/Symptom	Grade 0	Grade 1	Grade 2	Grade 3	Grade 4
Neuropathy-sensory	Normal	Loss of deep tendon reflexes or paresthesia (including tingling) but not interfering with function	Objective sensory loss or paresthesia (including tingling), interfering with function, but not interfering with activities of daily living	Sensory loss or paresthesia interfering with activities of daily living	Permanent sensory loss that interferes with function
Note: Peripheral Sensory Phenomena (PSP) can be a manifestation of the Mazzotti reaction					
Rigors, chills	None	Mild, requiring symptomatic treatment (e.g., blanket) or non-narcotic medication	Severe and/or prolonged, requiring narcotic medication	not responsive to narcotic medication	-
Fatigue (lethargy, malaise, asthenia)	None	Increased fatigue over baseline, but not altering normal activities	Moderate (e.g., decrease in performance status by 1 ECOG level or 20% Karnofsky or *Lansky*) or causing difficulty performing some activities	Severe (e.g., decrease in performance status by ≥2 ECOG levels or 40% Karnofsky or *Lansky*) or loss of ability to perform some activities	Bedridden or disabling
**OTHER SYSTEMIC TOXICITY**					
Anorexia	None	Loss of appetite	oral intake significantly decreased	Requiring IV fluids	Requiring feeding tube or parenteral nutrition
Nausea	None	Able to eat	oral intake significantly decreased	no significant intake, requiring IV fluids	-
'Bitter' mouth	None	Able to eat	oral intake significantly decreased	no significant intake, requiring IV fluids	-
Vomiting	None	1 episode in 24 hours over pretreatment	2-5 episodes in 24 hours over pretreatment	≥6 episodes in 24 hours over pretreatment; or need for IV fluids	Requiring parenteral nutrition; or physiologic consequences requiring intensive care; hemodynamic collapse
Diarrhea	None	Increase of < 4 stools/day over pre-treatment	Increase of 4-6 stools/day, or nocturnal stools	Increase of ≥7 stools/day or incontinence; or need for parenteral support for dehydration	Physiologic consequence requiring intensive care; or hemodynamic collapse
Abdominal pain or cramping	None	Mild pain not interfering with function	Moderate pain: pain or analgesics interfering with function, but not interfering with activities of daily living	Severe pain: pain or analgesics severely interfering with activities of daily living	Disabling
Cough	Absent	Mild, relieved by non-prescription medication	Requiring narcotic antitussive	Severe cough or coughing spasms, poorly controlled or unresponsive to treatment	-
Chest pain (non-cardiac and non-pleuritic)	None	Mild pain not interfering with function	Moderate pain: pain or analgesics interfering with function, but not interfering with activities of daily living	Severe pain: pain or analgesics severely interfering with activities of daily living	Disabling
Dyspnea (shortness of breath)	Normal	-	Dyspnea on exertion	Dyspnea at normal level of activity	Dyspnea at rest or requiring ventilator support
Palpitations	None	Present	-	-	-
Note: Grade palpitations only in the absence of a documented arrhythmia.					
Waistpain/Backache	None	Mild pain not interfering with function	Moderate pain: pain or analgesics interfering with function, but not interfering with activities of daily living	Severe pain: pain or analgesics severely interfering with activities of daily living	Disabling

Sign/Symptom	Grade 0	Grade 1	Grade 2	Grade 3	Grade 4
Neckpain	None	Mild pain not interfering with function	Moderate pain: pain or analgesics interfering with function, but not interfering with activities of daily living	Severe pain: pain or analgesics severely interfering with activities of daily living	Disabling
Bodily pain/aches	None	Mild pain not interfering with function	Moderate pain: pain or analgesics interfering with function, but not interfering with activities of daily living	Severe pain: pain or analgesics severely interfering with activities of daily living	Disabling
Dizziness/lightheadedness	None	Not interfering with function	Interfering with function, but not interfering with activities of daily living	Interfering with activities of daily living	Bedridden or disabling
Note: Grade only if unassociated with hypotension					
Constipation	None	Requiring stool softener or dietary modification or increased mobility	Requiring laxatives	Obstipation requiring manual evacuation or enema	Obstruction or toxic megacolon
Dyspepsia/heartburn	None	Mild	Moderate	Severe	-
Insomnia	Normal	Occasional difficulty sleeping not interfering with function	Difficulty sleeping interfering with function, but not interfering with activities of daily living	Frequent difficulty sleeping, interfering with activities of daily living	-
Note: This toxicity is graded when insomnia is related to treatment. (Sleepless for no reason). If pain or other symptoms interfere with sleep do NOT grade as insomnia.					
Bone pain	None	Mild pain not interfering with function	Moderate pain: pain or analgesics interfering with function, but not interfering with activities of daily living	Severe pain: pain or analgesics severely interfering with activities of daily living	Disabling
Catarrh	Absent	Present	-	-	-
Earache (otalgia)	None	Mild pain not interfering with function	Moderate pain: pain or analgesics interfering with function, but not interfering with activities of daily living	Severe pain: pain or analgesics severely interfering with activities of daily living	Disabling
Note: It is essential to determine that otalgia is not present pretreatment					
Toothache	None	Mild pain not interfering with function	Moderate pain: pain or analgesics interfering with function, but not interfering with activities of daily living	Severe pain: pain or analgesics severely interfering with activities of daily living	Disabling
Note: It is essential to determine that toothache is not present pretreatment					
Weight gain	< 5%	5 - <10%	10 - <20%	≥ 20%	-
Weight loss	< 5%	5 - <10%	10 - <20%	≥20%	-
Erectile impotence	Normal	Mild (erections impaired but satisfactory)	Moderate (erections impaired, unsatisfactory for intercourse)	no erections	-
Libido	Normal	Decrease in interest	severe loss of interest	-	-
Dysmenorrhea	None	Mild pain not interfering with function	Moderate pain: pain or analgesics interfering with function, but not interfering with activities of daily living	Severe pain: pain or analgesics severely interfering with activities of daily living	Disabling
Irregular menses (change from baseline)	Normal	Occasionally irregular or lengthened interval, but continuing menstrual cycles	very irregular, but continuing menstrual cycles	Persistent amenorrhea	-
**MAZZOTTI REACTION CRITERIA - OCULAR REACTIONS**					

Sign/Symptom	Grade 0	Grade 1	Grade 2	Grade 3	Grade 4
Ocular discomfort	Normal	mild: not interfering with function	Moderate: interfering with function, but not interfering with activities of daily living	Interfering with activities of daily living	-
Ocular itching	Normal	mild: not interfering with function	Moderate: interfering with function, but not interfering with activities of daily living	Interfering with activities of daily living	-
Tearing (watery eyes)	None	mild: not interfering with function	Moderate: interfering with function, but not interfering with activities of daily living	Interfering with activities of daily living	-
Vision- photophobia	Normal	-	Symptomatic and interfering with function, but not interfering with activities of daily living	Symptomatic and interfering with activities of daily living	-
Visual acuity	Un-changed or 6/4-6/6	Loss of 1 line	Loss of 2 lines	Loss of 3 lines	Loss of >3 lines
Anterior Segment inflammation					
Conjunctivitis	None	-	Hyperaemia	-	Chemosis
Limbitis-vascular changes	None	-	Dilated capillaries	-	Limbal oedema
Limbitis-globular infiltrates	None	1-5	6-10	11-20	>20
Corneal punctate opacities	None	1-5	6-10	11-20	>20
Anterior uveitis					
No of cells/field *	None	1-10	11-20	21-40	>40
Flare**	None	Seen with no filter	Seen with filter 1	Seen with filter 2	Plasmoid aqueous
Note: * Light beam at 45 degrees; slit 2mm high by 0.2mm wide. ** Light beam at 45 degrees; slit 2mm high by 0.1mm wide. Filters 1 and 2 are neutral density filters in-built into the optical column of the slit lamp					
Posterior segment					
Optic neuritis (colour film)					
Hyperaemia	None	Sectorial	Overall	-	-
Swelling	None	-	-	Sectorial	Overall
Angiographic leakage					
Within disc margin	None	Sectorial	Overall	-	-
Beyond disc margin	None	-	-	Sectorial	Overall
Optic atrophy					
Colour film	None	Linear nerve fibre loss	Sectorial atrophy	Overall atrophy	Atrophy with pigment
Red free	None	Linear nerve fibre loss	Sectorial fibre loss	Total loss	Total loss with pigment
Pigment Epithelial Atrophy					
Distribution	None	Temporal to macula	More than temporal	Continuous round macula	Whole of macula
Intensity	None	RPE mottling only	RPE mottling with <50% atrophy	RPE mottling with ≥50% atrophy	RPE mottling with hypertrophy
Other onchocercal lesions					
Cotton wool spots	Absent	Present	-	-	-
Vasculitis	Absent	Present	-	-	-
Haemorrhage	Absent	Present	-	-	-
Note: Once an event has occurred the score is retained at subsequent visits whether the lesion persists or not.					
**OTHER OCULAR TOXICITY**					
Ocular discharge	None	Present	-	-	-
Vision- blurred vision	Normal	-	Symptomatic and interfering with function, but not interfering with activities of daily living	Symptomatic and interfering with activities of daily living	-

Sign/Symptom	Grade 0	Grade 1	Grade 2	Grade 3	Grade 4
Vision- flashing lights/floaters	Normal	Mild, not interfering with function	Symptomatic and interfering with function, but not interfering with activities of daily living	Symptomatic and interfering with activities of daily living	-
Vision- night blindness (nyctalopia)	Normal	Present but asymptomatic	Symptomatic and interfering with function, but not interfering with activities of daily living	Symptomatic and interfering with activities of daily living	-
**ALLERGIC REACTION commonly to FLUORESCEIN**					
Allergic reaction/ hypersensitivity (including drug fever)	None	Transient rash, drug fever < 38°C (<100.4°F)	urticaria, drug fever ≥ 38°C (≥100.4°F), and/or asymptomatic bronchospasm	Symptomatic bronchospasm, requiring parenteral medication(s), with or without urticaria; allergy-related edema/angioedema	Anaphylaxis
Sneezing	Absent	Present	-	-	-
Dry cough, no bronchospasm	Absent	Present	-	-	-
Urticaria (hives, welts, wheals)	None	Requiring no medication	requiring PO or topical treatment or IV medication or steroids for <24 hours	requiring IV medication or steroids for ≥24 hours	-
Note: Also anorexia, nausea, vomiting , rigors, chills					
**ELECTROCARDIOGRAPHY**					
Conduction abnormality/ Atrioventricular heart block	None	Asymptomatic, not requiring treatment (e.g., Mobitz type I second-degree AV block, Wenckebach)	Symptomatic, but not requiring treatment	symptomatic and requiring treatment (e.g., Mobitz type II second-degree AV block, third-degree AV block)	Life-threatening (e.g., arrhythmia associated with CHF, hypotension, syncope, shock)
Nodal/junctional arrhythmia/dysrhythmia	None	Asymptomatic, not requiring treatment	symptomatic, but not requiring treatment	symptomatic and requiring treatment	Life-threatening (e.g., arrhythmia associated with CHF, hypotension, syncope, shock)
Palpitations	None	Present	-	-	-
Note: Grade palpitations only in the absence of a documented arrhythmia.					
Prolonged QTc interval (QTc > 0.48 seconds)	None	Asymptomatic, not requiring treatment	symptomatic, but not requiring treatment	symptomatic and requiring treatment	Life-threatening (e.g., arrhythmia associated with CHF, hypotension, syncope, shock)
Sinus bradycardia	None	Asymptomatic, not requiring treatment	symptomatic, but not requiring treatment	symptomatic and requiring treatment	Life-threatening (e.g., arrhythmia associated with CHF, hypotension, syncope, shock)
Sinus tachycardia	None	Asymptomatic, not requiring treatment	symptomatic, but not requiring treatment	symptomatic and requiring treatment of underlying cause	-
Supraventricular arrhythmias (SVT/atrial fibrillation/ flutter)	None	Asymptomatic, not requiring treatment	symptomatic, but not requiring treatment	symptomatic and requiring treatment	Life-threatening (e.g., arrhythmia associated with CHF, hypotension, syncope, shock)
Vasovagal episode	None	-	present without loss of consciousness	present with loss of consciousness	-
Ventricular arrhythmia (PVCs/bigeminy/trigeminy/ ventricular tachycardia)	None	Asymptomatic, not requiring treatment	symptomatic, but not requiring treatment	symptomatic and requiring treatment	Life-threatening (e.g., arrhythmia associated with CHF, hypotension, syncope, shock)
Peaking of T wave	None	Mild-moderate	Marked	-	-
Cardiac- ischemia/infarction	None	Non-specific T-wave flattening or changes	Asymptomatic, ST- and T- wave changes suggesting ischemia	angina without evidence of infarction	acute myocardial infarction
**HAEMATOLOGICAL**					
G6PD	Normal	Partial defect	Total defect	-	-
Hemoglobin (Hgb)	WNL	10-<25% reduction	25-<50% reduction	50-<75% reduction	≥75%
Leukocytes (total WBC)	WNL	$< 2{\cdot}8 – 2{\cdot}3 \times 10^9$ /L	$< 2{\cdot}3$ -$1{\cdot}8 \times 10^9$ /L	$<1{\cdot}8$ - $1{\cdot}3 \times 10^9$ /L	$< 1{\cdot}3 \times 10^9$ /L
	WNL	$> 11{\cdot}3$-$19{\cdot}0 \times 10^9$ /L	$>19{\cdot}0$-$38{\cdot}0 \times 10^9$ /L	$>38{\cdot}0$-$57{\cdot}0 \times 10^9$ /L	$> 57{\cdot}0 \times 10^9$ /L

Sign/Symptom	Grade 0	Grade 1	Grade 2	Grade 3	Grade 4
Note: Contributory factors include **Mazzotti** reaction (leucocytosis), coincidental infection (exclude) drug effect					
Neutrophils/granulocytes (ANC/AGC)	WNL	<0·476-0·376 10^9 /L	<0·376-0·276 10^9 /L	<0·276-0·176 10^9 /L	<0·176 10^9 /L
	WNL	>6·102-12·204 10^9 /L	>12·204-24·409 10^9 /L	>24·409-36·614 10^9 /L	>36614 10^9 /L
Note: Contributory factors include **Mazzotti** reaction (neutrophilia), coincidental infection (exclude) drug effect					
Lymphocytes	WNL	<0·980-0·880 10^9 /L	<0·880 -0·780 10^9 /L	<0·780-0·680 10^9 /L	<0·680 10^9 /L -
	WNL	>7·571-15·142 10^9 /L	>15·132-30·285 10^9 /L	>30·285-45·429 10^9 /L	>45·429 10^9 /L
Note: Contributory factors include **Mazzotti** reaction (initial lymphopenia, lymphocytosis), coincidental infection (exclude) drug effect					
Eosinophils	WNL	<LLN - 0·83 x LLN	<0·83 - 0·65 x LLN	<0·65 - 0·47 x LLN	<0·47 x LLN
(Any normal range applies)		>ULN - 2 x ULN	>2 - 4 x ULN	>4 - 6 x ULN	>6 x ULN
Note: Contributory factors include **Mazzotti** reaction (initial eosinopenia, eosinophilia), coincidental infection (exclude) drug effect					
Eosinophils	WNL	<0·028-0·023 10^9 /L	<0·023-0·018 10^9 /L	<0·018-0·013 10^9 /L	<0·013 10^9 /L
(normal range = 1-11 %)	WNL	>1·243-2·486 10^9 /L	>2·486-4·972 10^9 /L	>4·972-7·458 10^9 /L	>7·458 10^9 /L
Note: Contributory factors include **Mazzotti** reaction (initial eosinopenia, eosinophilia), coincidental infection (exclude) drug effect					
Eosinophils	WNL	<0·028-0·023 10^9 /L	<0·023-0·018 10^9 /L	<0·018-0·013 10^9 /L	<0·013 10^9 /L
(normal range = 1-6 %)	WNL	>0·678-1·356 10^9 /L	>1·356-2·712 10^9 /L	>2·712-4·068 10^9 /L	>4·068 10^9 /L
Note: Contributory factors include **Mazzotti** reaction (initial eosinopenia, eosinophilia), coincidental infection (exclude) drug effect					
Platelets	WNL	< LLN - <75·0 x 10^9 /L	≥50·0 - < 75·0 x 10^9 /L	≥10·0 - < 50·0 x 10^9 /L	< 10·0 x 10^9 /L
Hemolysis (e.g., immune hemolytic anemia, drug-related hemolysis, other)	None	Only laboratory evidence of hemolysis [e.g., direct antiglobulin test (DAT, Coombs') schistocytes]	Evidence of red cell destruction and ≥ 2gm decrease in hemoglobin, no transfusion	Requiring transfusion and/or medical intervention (e.g., steroids)	Catastrophic consequences of hemolysis (e.g., renal failure, hypotension, bronchospasm, emergency splenectomy)
**BIOCHEMICAL**					
Hyponatremia	WNL	<LLN – 130 mmol/L	-	120 - <130 mmol/L	<120 mmol/L
Hypernatremia	WNL	> ULN - 150 mmol/L	>150 - 155 mmol/L	>155 - 160 mmol/L	>160 mmol/L
Hypokalemia	WNL	<LLN - 3·0 mmol/L	-	2·5 - <3·0 mmol/L	<2·5 mmol/L
Hyperkalemia	WNL	> ULN - 5·5 mmol/L	> 5·5 - 6·0 mmol/L	> 6·0 - 7·0 mmol/L	> 7·0 mmol/L
Bicarbonate	WNL	< LLN - 16 mEq/dl	11 - 15 mEq/dl	8 - 10 mEq/dl	<8 mEq/dl
Hyperuricemia	WNL	> ULN - ≤ 590 micromol/L without physiologic consequences	-	>ULN - ≤ 590 micromol/L with physiologic consequences	> 590 micromol/L
Note: Also consider Renal failure, Creatinine, Potassium.					
Creatinine	WNL	> ULN - 1·5 x ULN	> 1·5 - 3·0 x ULN	> 3·0 - 6·0 x ULN	> 6·0 x ULN
SGPT (ALT) (serum glutamic pyruvic transaminase)	WNL	> ULN - 2·5 x ULN	> 2·5 - 5·0 x ULN	> 5·0 - 20·0 x ULN	> 20·0 x ULN
Note: Elevations occur as part of the **Mazzotti** reaction but rarely reach 5 x ULN and usually last for less than 30 days					
SGOT (AST) (serum glutamic oxaloacetic transaminase)	WNL	> ULN - 2·5 x ULN	> 2·5 - 5·0 x ULN	> 5·0 - 20·0 x ULN	> 20·0 x ULN
Note: Elevations occur as part of the **Mazzotti** reaction but rarely reach 5 x ULN and usually last for less than 30 days					
GGT (γ - Glutamyl transpeptidase)	WNL	> ULN - 2·5 x ULN	> 2·5 - 5·0 x ULN	> 5·0 - 20·0 x ULN	> 20·0 x ULN
Note: Elevations occur as part of the **Mazzotti** reaction but rarely reach 5 x ULN and usually last for less than 30 days					
LDH (Lactate dehydrogenase)	WNL	> ULN - 2·5 x ULN	> 2·5 - 5·0 x ULN	> 5·0 - 20·0 x ULN	> 20·0 x ULN
Note: Elevations occur as part of the **Mazzotti** reaction but rarely reach 5 x ULN and usually last for less than 30 days					
Alkaline phosphatase	WNL	> ULN - 2·5 x ULN	> 2·5 - 5·0 x ULN	> 5·0 - 20·0 x ULN	> 20·0 x ULN

Sign/Symptom	Grade 0	Grade 1	Grade 2	Grade 3	Grade 4
Total Bilirubin	WNL	> ULN - 1·5 x ULN	> 1·5 - 3·0 x ULN	> 3·0 - 10·0 x ULN	> 10·0 x ULN
Hypoalbuminemia	WNL	<LLN - 30 g/L	≥20 - <30 g/L	<20 g/L	-
Hypoglycemia	WNL	<LLN - 3·0 mmol/L	2·2 - < 3·0 mmol/L	1·7 - < 2·2 mmol/L	< 1·7 mmol/L
Hyperglycemia	WNL	> ULN - 8·9 mmol/L	> 8·9 - 13·9 mmol/L	> 13·9 - 27·8 mmol/L	> 27·8 mmol/L or ketoacidosis
Hypercholesterolemia	WNL	> ULN - 7·75 mmol/L	> 7·75 - 10·34 mmol/L	>10·34 - 12·92 mmol/L	> 12·92 mmol/L
Hypertriglyceridemia	WNL	> ULN - 2·5 x ULN	> 2·5 - 5·0 x ULN	> 5·0 - 10 x ULN	> 10 x ULN
**URINE**					
Protein	Negative or Trace	1 +	2 + to 3 +	4 +	Nephrotic syndrome
Blood (in the absence of vaginal bleeding)	None (Negative on urinalysis via multistix)	Microscopic only, ≥ 4 to <50 RBC/HPF	Intermittent gross bleeding	Persistent gross bleeding or clots; may require catheterization or instrumentation or transfusion	Open surgery or necrosis or deep bladder ulceration
Specific Gravity	1·005-1·030	NA	NA	NA	NA
Leukocyte Esterase	Negative	Positive AND ≥ 6 WBC/HPF	NA	NA	NA
Nitrite	Negative	Positive AND ≥ 6 WBC/HPF	NA	NA	NA
pH	5·0 - 9·0	NA	NA	NA	NA
GIucose	Negative	Trace to 2+	3- 4+	NA	NA
Ketones	Negative	Trace to moderate	Large	NA	NA
Bilirubin	Negative	Small to moderate	Large	NA	NA
Urobilinogen (µmol/L)	<1	1·0-2·0	4-8	NA	NA
White blood cells	<5 /HPF	≥ 6 WBC/HPF	NA	NA	NA
Red Blood Cells	0-3/HPF	≥ 4 to <50 RBC/HPF	NA	NA	NA

Table S 3: Grading of visual fields abnormalities measured via FDT perimetry

Grade	Mean Deviation (MD) value
0<br>(normal)	MD value is positive OR negative AND<br>Mean deviation is ≥ 95% of FDT fields of subjects of that age with normal vision (no percentile indicated after the MD value)
1<br>(mild)	MD value is negative AND<br>The probability is <5% that the overall sensitivity of the subject is in the range of ≥95% of the people with normal vision of that age (P<5% indicated after the MD value)
2<br>(moderate)	MD value is negative AND<br>The probability is <2% that the overall sensitivity of the subject is in the range of ≥95% of the people with normal vision of that age (P<2% indicated after the MD value)
3<br>(severe)	MD value is negative AND<br>The probability is <1% that the overall sensitivity of the subject is in the range of ≥95% of the people with normal vision of that age (P<1% indicated after the MD value)
4<br>(very severe)	MD value is negative AND<br>The probability is <0.5% that the overall sensitivity of the subject is in the range of ≥95% of the people with normal vision of that age (P<0.5% indicated after the MD value)

Table S 4: Grading of colour vision with PV16

Grade	Criteria
Normal (grade 0)	< four (4) crossings if there is **NO** definitive axis (i.e. the majority of crossings are not parallel to the protan, deutan or tritan axes on the circular result reporting form)
Abnormal<br>(mild, grade 1)	4 - 10 crossings<br>OR<br><4 crossings with a definitive axes (i.e. all parallel to one of the three axes defined on the result reporting sheet (protan, deutan or tritan)
Abnormal<br>(moderate, grade 2)	Not defined
Abnormal<br>(severe, grade 3)	> 10 crossings

Table S 5: Grading of laboratory events not included in other grading scales provided

	Grade				
**Paramter**	**0**	**1**	**2**	**3**	**4**
Platelet increase	WNL	> ULN<br>– 850 x 10^9 /L	> 850 x 10^9 /L<br>– 1500 x 10^9 /L	> 1500 x 10^9 /L	NA
Monocyte increase	WNL	> 1·13 x 10^9 /L<br>– 2·26 x 10^9 /L	> 2·26 x 10^9 /L<br>– 4·52 x 10^9 /L	> 4·52 x 10^9 /L<br>– 9·04 x 10^9 /L	> 9·04 x 10^9 /L
Basophil increase	WNL	> 0,1 x 10^9 /L<br>– 0·2 x 10^9 /L	> 0,2 x 10^9 /L<br>– 0·4 x 10^9 /L	> 0,4 x 10^9 /L<br>– 0·8 x 10^9 /L	> 0,8 x 10^9 /L

1.4 Mazzotti reactions

1.4.1 Overview of Mazzotti reactions

Common systemic clinical manifestations of the Mazzotti reaction include pruritus, rash, lymphadenitis, headache, myalgia, arthralgia, hypotension, fever, and swelling of the face and limbs.[1] Ocular events include epiphora, photophobia, conjunctival injection, limbitis, anterior uveitis, chorioretinitis, and optic neuritis.[2] The clinical laboratory changes involve the peripheral blood leukocytes, AST, ALT and sometimes LDH, and GGT; microfilariae also appear in blood, urine, and other body fluids. The eosinophils exhibit the most prominent changes. There may be an initial eosinopenia followed several days later by a marked increase above pretreatment levels. A complete disappearance of eosinophils from the peripheral blood is the laboratory hallmark of a severe reaction. Lymphocyte counts may fall initially, followed by lymphocytosis, but not to the same extent as with eosinophils. Leukocytosis with neutrophilia is less common. Elevations in liver enzymes occur, but rarely exceed grade 2 and usually normalize by day 30. Levels of bilirubin and AP are usually unchanged. Proteinuria may occur. Although the laboratory changes *per se* have little clinical significance, they give indirect evidence of the death of microfilariae and may even indicate the speed and severity of the reaction to the event.[3] The factors that govern the Mazzotti reaction include the intensity of infection,[4] the dose regimen, and the microfilaricide used. These determine the onset, evolution, reaction severity, extent of the laboratory changes, and whether the reaction is mono- or biphasic. A notable exception occurs in subjects with hyperreactive onchodermatitis (Sowda),[5] where severe, predominantly cutaneous adverse effects occur even with very low skin microfilarial counts.

For a given intensity of infection, the severity of the Mazzotti reaction to ivermectin is independent of dose within the range of 150 to 800 µg/kg (approximately 9 mg to 48 mg for weights between 45 kg and 64 kg).[6] Dangerous and alarming reactions result from the simultaneous occurrence of severe reactions in multiple systems.[7] This phenomenon is rare with ivermectin. Biphasic reactions, characterized by a marked

recrudescence of cutaneous or lymph node symptoms or the development of an acute febrile polyarthritis,[8] several days after the initial reaction, have also not been observed with ivermectin.

1.4.2 Coding of adverse events characterized as Mazzotti reactions

The Medical Dictionary for Regulatory Agencies (MedDRA), the standard for coding of investigator verbatims of adverse events (AE) into 'preferred terms' and their assignment to system organ classes, results for common types of Mazzotti reactions (e.g. swelling, pruritus, pain) into distribution across different system organ classes and preferred terms. This does not facilitate comparison of the frequency of Mazzotti reactions after different treatments. Therefore, AEs characterized as Mazzotti reactions were coded and grouped into reaction clusters and reaction groups with a dictionary specifically designed for Mazzotti reactions based on the experience at OCRC (Table S 6).

Table S 6: Dictionary for coding signs and symptoms of onchocerciasis and AEs characterized as Mazzotti reactions into reaction clusters and groups

Reaction Cluster	Reaction Group	Sign/Symptom
**Clinical Mazzotti reactions**	Dermal	Pruritus
		Rash
	Glandular	Lymph node pain
		Gland pain
		Lymph node tenderness
	Musculo skeletal	Arthralgia
		Myalgia
	Febrile	Headache
		Pyrexia
	Cardiovascular	Orthostatic hypotension
		Severe symptomatic postural hypotension (SSPH)
		Heart rate standing increased (</=100/min)
		Postural tachycardia
		Supine hypotension
		Heart rate increased lying (</=100/min)
		Supine tachycardia
	Asthenia	Asthenia
		Fatigue
		Lethargy
		Malaise
	Back pain	Back pain
		Waist pain
	Rigors/Chills	Rigors<br>Chills
	Dizziness	Dizziness<br>light headedness
		Dizziness postural
	Nausea	Nausea postural
	Pain (other than back pain, waist pain, extremities)	Pain (neckpain, bodily pain/aches)
	Peripheral sensory phenomena	Peripheral sensory phenomena (tingling, stinging, burning sensation, paraesthesia, heaviness, numbness, hot flushes)
	Other sensory phenomena	Other sensory phenomena
	Swelling	Brawny oedema
		Face oedema
		Joint swelling
		Lymph node oedema
		Other oedema
	Tachypnea	Tachypnea (Increased respiratory rate)
	Urticaria	Urticaria
**Laboratory Mazzotti Reactions**	Haematology	Leukocytopenia (CS or not)
		Leukocytosis (CS or not)
		Eosinopenia (CS or not)
		Eosinophilia (CS or not)
		Lymphopenia (CS or not)
		Lymphocytosis (CS or not)
		Neutrophilia (CS or not)

Reaction Cluster	Reaction Group	Sign/Symptom
	Serum Biochemistry	SGOT_AST > ULN (CS or not)
		SGPT_ALT > ULN (CS or not)
		γ-GT > ULN (CS or not)
		AP > ULN (CS or not)
		LDH > ULN (CS or not)
	Urine	Proteinuria (CS or not)
		Microscopic haematuria (CS or not)
**Ocular reactions**	Conjunctiva	Conjunctivitis
		Limbitis Vascular changes
		Limbitis Globular infiltrates
	Cornea	Punctate opacities
	Iris	No of cells/field
		Flare
		Iris
	Optic disk	Hyperaemia
		Swelling
		Aspect of optic disk
	Peripheral Retina	Distribution of atrophy<br>Intensity of Retinal Pigmental Epithelial mottling and % of atrophy
		Retinitis
		Cotton wool spots
		Vasculitis
		Haemorrhage
	Eye Pain	Eye pain
	Eye Pruritus	Eye pruritus
	Eyelid Swelling	Eyelid oedema
	Vision	Photophobia
		Visual acuity
		Visual field change
		Colour vision
		Blurred vision
	Other ocular	Tearing/watery eyes
	Mazzotti reactions	Ocular discomfort

CS – clinically significant.

1.5 Sample size calculation

The distribution and mean pre-treatment skin microfilariae levels which impact the skin microfilariae levels 12 months after ivermectin treatment could not be predicted during study planning since they depend on a number of unknown factors including the level of endemicity in the villages from which participants would be recruited as well as the age and thus life time risk of infection of each participant and area and individual treatment history.

Consequently, sample size calculations to show a 50% difference in arithmetic mean SmfD 12 months after treatment between the two treatment arms took into account data obtained 12 months after ivermectin treatment from two different historical scenarios: the first community study of ivermectin[9] (raw data provided by Dr. H. Remme, 32·49 ± 2·35 mf/snip) and a study conducted at the OCRC (4·01 ± 2·41 mf/mg skin). We assumed that the Month 12 mean values in the ivermectin arm would be one or the other, with the Month 12 value in the moxidectin arm being half that in the ivermectin arm (16.24 mf/snip and 2.0 mf/mg, respectively) and with the estimate of the common standard deviation being that in the historical scenario (2.35 mf/snip or 2.41 mf/mg, respectively).

Sample size calculation was conducted for both scenarios for the case of a 2-sided test, type I error of 0.05 and power of 90% based on a student t-test and $\log_e(y+1)$ transformed data, taking into account the randomization ratio of 2:1 moxidectin to ivermectin, previously decided upon in view of safety data collection. For the two historical scenarios this yielded a total sample size of 159 (106:53, moxidectin:ivermectin) and 276 (184:92), respectively. Assuming that only 65% of enrolled participants would be available for the 12 months visit and taking into account the planned block size of 6 for randomization, the sample sizes were 246 and 426, respectively. This was judged not to provide a sufficient number of participants for the safety-related part of the primary study objective. The planned sample size was increased to approximately 1000 moxidectin : 500 ivermectin treated participants, which, assuming 65% evaluability (650 moxidectin treated, 325 ivermectin treated participants having Month 12 SmfD data), yielded >99% power to detect a ≥50% treatment difference.

1.6 Statistical analysis

1.6.1 Statistical methods for primary efficacy endpoint

SmfD were logarithmically transformed ($y = \log_e(SmfD+1)$) before analysis. Back transformation was carried out by directly exponentiating least squares means (LSM) as well as the lower and upper confidence limit and subtracting 1.

SmfD 12 months post-treatment was compared with a mixed-effects model as described in the manuscript including sex*treatment and LoI*treatment interactions. When no interactions were detected ($p > 0.05$), the interaction term was not considered and the main effects model was used for the primary analysis. When an interaction was detected, adjusted (least square) means were calculated for each combination of the treatments and the sex and LoI categories and compared pairwise to evaluate for which level of the stratification variable there was a statistically significant difference between treatments. For this analysis, no imputations for missing data were made. The mixed model is valid in the presence of missing data, if the missing data can be considered missing at random which was the case: in each treatment group 3% of treated participants did not have a month 12 evaluation.

The model fit was assessed in 2 ways: (1) comparison with a model with intercept only. Here the log-likelihood ratio test was used. (2) choice of the variance-covariance matrix for random effects and for repeated effects was performed by using AIC and BIC. The lower AIC represented the best matrix to choose.

An R-square can be approximated by focusing on percent reduction in variability due to the model where

$$R\text{-}square = 1 - SSE/SSTOT$$

with SSE the sum of the squared residuals from the model and SSTOT the sum of squared residuals from an intercept only model:

Source	Sum of Squares
Residual Full Model	784.50645
Residual Intercept Model	1682.670887
R square	0.533773

By default the structure of the variance – covariance matrix for the repeated measurements part of the model was unstructured (UN). When UN failed to converge, other variance – covariance matrix structures including Toeplitz (TOEP) compound symmetry (CS), variance components (VC) and first-order autoregressive (AR (1)) were considered. Akaike Information Criteria (AIC) was used to choose between them. Two-way interactions were evaluated and analysed when necessary as described for the primary efficacy variable.

As per protocol, a non-parametric model was fitted to the data. It was performed by first transforming the outcome data to Savage ranks and analyzing these transformed data with the same mixed model described in the manuscript for the primary efficacy outcome. As per peer reviewer recommendations, sensitivity analyses were conducted:

- a linear fixed effects model with baseline SmfD, treatment, sex, LoI, treatment*sex and treatment*LoI interactions, and site as fixed effects,
- a linear mixed model with % change in skin microfilarial density at 12 months from baseline the outcome variable. Baseline SmfD, treatment, sex, LoI, treatment*sex, and treatment*LoI interactions were fixed effects and site the random effect.

1.6.2 Analysis populations

The modified intent-to-treat (mITT) population (population for primary efficacy and secondary SmfD based analysis and safety analysis) was defined as all participants who received a single dose of study drug.

The secondary population for efficacy analysis was the efficacy-modified intent-to-treat (e-mITT) population defined as all participants who received treatment and for whom skin microfilariae counts were obtained at baseline and month 12. Since the e-mITT population constituted around 98% of the mITT population, only the results for the mITT population are provided.

1.7 Overview of protocol amendments

Study initiation occurred under Amendment 1.

Amendment 2 incorporated clarifications provided previously to the investigators via sponsor memoranda, i.e.

- prohibited prior and concomitant anthelminthics were anti-nematodal drugs,
- protocol-scheduled laboratory determinations should be conducted only with sponsor-provided equipment and back-up equipment, samples preserved if neither was functioning and that other methods

- should be used only in the absence of functioning sponsor-provided equipment when the investigator needs the results for safety reasons
- randomization of participants with 20 mf/mg.
- biological samples should be destroyed only after verification of the protocol-specified data by the laboratory head and investigator or designate.

and added immunochromatographic tests during follow up for participants with positive tests for LF at baseline.

Amendment 3 was instituted after completion of enrolment in all sites and completion of follow up of all participants enrolled in Liberia to

- update sponsor and sponsor-related information
- eliminate the Month 18 visit for all participants whose Month 18 visit occurred after 31 December 2011 or approval of the amendment by the Ethics Committees in DRC and Ghana and update or clarify all related information and provisions

Furthermore, Amendment 3

- included clarifications which investigators been previously provided with via Sponsor Memoranda (provision of best estimate for number of punctate opacities when ophthalmologists considered accurate counting impossible, application of adverse event criteria to laboratory and ECG findings)
- updated the section on Clinical Experience with moxidectin to include the results of analyses completed for the healthy volunteer studies and the Phase 2 study.

There were no changes in the objectives, or planned sample size or endpoints or the planned analysis of the primary efficacy outcome, i.e. mixed effect models including baseline skin microfilariae density, treatment group, visit, treatment by visit, and stratification variables sex and center.

Before unblinding, center was selected as the random effect for the mixed effect model and the level of infection added to the factors in the mixed effect model.

2 Results

2.1 Baseline characteristics

2.1.1 Skin microfilariae

Table S 7: Skin microfilariae density in subgroups defined by level of infection at baseline and presence of palpable nodules pre-treatment

	8 mg moxidectin	Ivermectin
Mean±SD mf/mg skin in those with <20 mf/mg	13·9±2·9	14·1±2·9
Mean±SD mf/mg skin in those with ≥20 mf/mg	48·9±30·9	53·0±30·7
No (%) without palpable nodules[a]	551 (56·3)	279 (56·5)
No (%) with > 4 nodule sites	44 (4·5)	26 (5·3)

[a]Palpable nodules were not an inclusion criterion and are not necessarily onchocercal.[10-12]

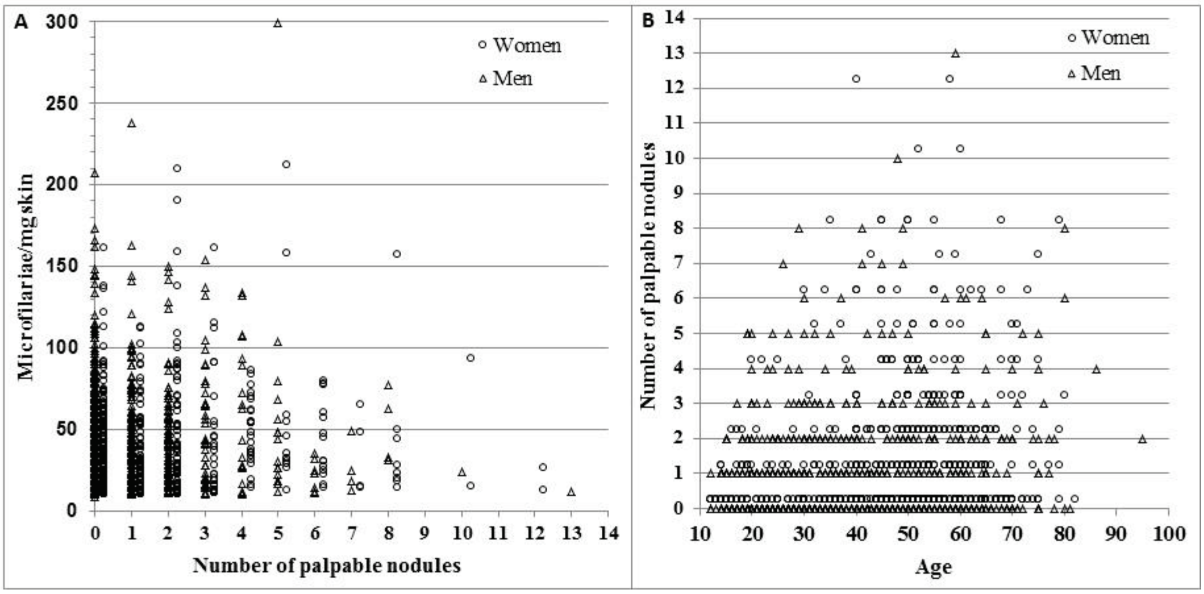

Figure S 1 A: Number of nodules palpated pre-treatment vs pre-treatment skin microfilariae density by sex of participants; B: Participant age vs. number of palpable nodules pre-treatment.

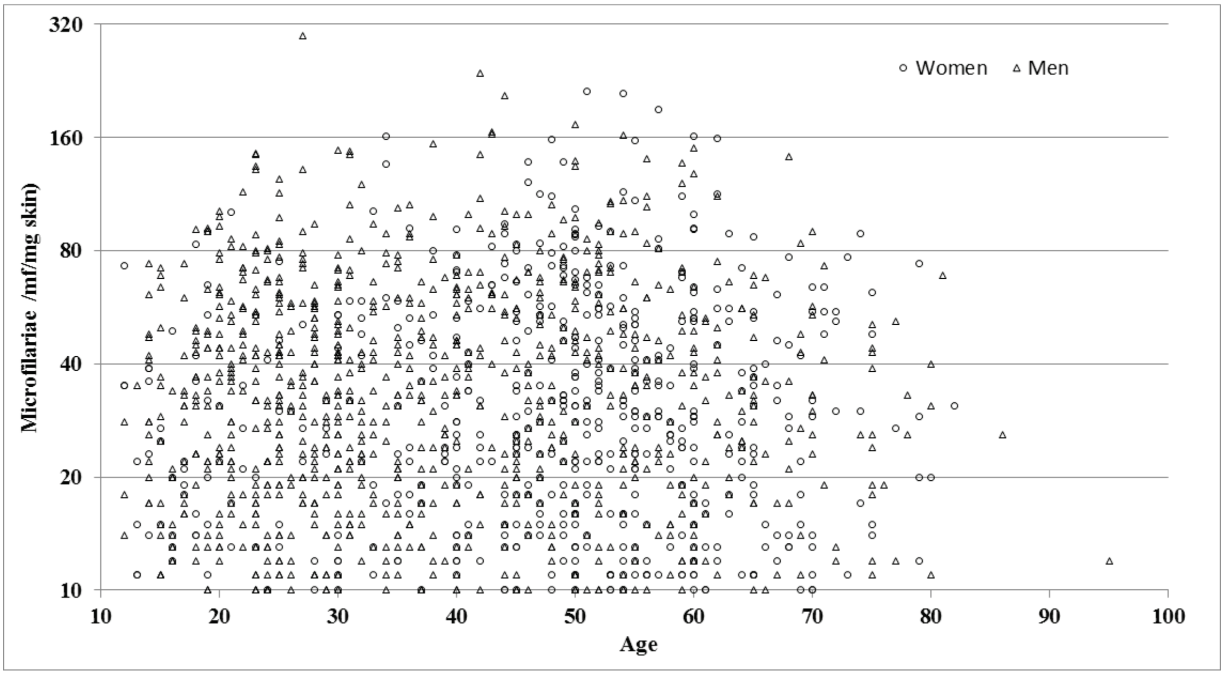

Figure S 2 Skin microfilariae density pre-treatment by age and sex

2.1.2 Results of ICT testing pre-treatment

ICT were conducted in Liberia and DRC where LF endemicity was unknown prior to this study. The area from which participants were recruited in Ghana is known not to be LF endemic.

DRC - Nord-Kivu: All 487 tested were ICT negative.

DRC - Nord-Ituri: 466/470 tested were ICT negative. None of three ICT positive who were tested for microfilariae in the blood had detectable levels.

Liberia: 30/299 tested were ICT positive. None had detectable levels of microfilariae in the blood.

2.1.3 Intestinal helminth infection pre-treatment

Table S 8: Intestinal helminth infections at baseline by site

	Nord-Kivu, DRC				Nord-Ituri, DRC				Lofa County, Liberia			Nkwanta district, Ghana			
**Age**	<18		≥18		<18		≥18		<18	≥18		<18		≥18	
**N with data**	3		451		26		446		0	283		46		183	
	n	%	n	%	n	%	n	%		n	%	n	%	n	%
**Not infected**	0	0	106	24	11	42	191	43		186	66	13	28	77	42
**Hookworm**	3	100	313	69	10	38	214	48		87	31	32	70	103	56
**Roundworm**	1	33	15	3	1	4	15	3		9	3	1	2	2	1
**Schistosoma**	1	33	103	23	9	35	85	19		13	5	2	4	1	1
**Enterobius**	0	0	0	0	0	0	2	0		0	0	0	0	0	0
**Tapeworm**	0	0	0	0	1	4	0	0		0	0	0	0	0	0
**Strongyloides**	0	0	0	0	0	0	1	0		3	1	0	0	0	0
**Whipworm**	0	0	11	2	0	0	3	1		0	0	1	2	4	2
**1 species**	1	33	256	57	10	38	193	43		82	29	30	65	102	56
**2 species**	2	67	81	18	4	15	59	13		15	5	3	7	4	2
**3 species**	0	0	8	2	1	4	3	1		0	0	0	0	0	0

2.2 Efficacy data

2.2.1 Primary efficacy endpoint (SmfD 12 months post-treatment) analysis with protocol-specified mixed effects model

Table S 9: Primary efficacy endpoint: Descriptive statistics and protocol-specified mixed-effects model analysis

Descriptive statistics SmfD across all participants

Time point	Treatment Group	N	AM (SD)	AM 95% CI	Median (1Q,3Q)	Min., Max.	GM ([95% CI]
BL*	Moxidectin	978	38·8 (30·5)	[36·9,40·8]	31·0 (18·0, 49·0)	9·0, 299·0	30·8 [29·5,32·1]
	Ivermectin	494	41·2 (31·3)	[38·4,43·9]	32·0 (17·0, 56·0)	10·0, 238·0	32·2 [30·2,34.3]
M12	Moxidectin	947	1·2 (3·0)	[1·0,1·4]	0·0 (0·0,1·0)	0·0, 28·0	0·5 [0·5,0·6]
	Ivermectin	480	10·0 (12·9)	[8·8,11·1]	5·0 (1·0,13·0)	0·0, 77·0	5·0 [4·4,5·7]

*p-value = 0.010 for comparison of treatment groups obtained from linear model where SmfD at baseline was the outcome variable with treatment, baseline level of infection (LoI, <20 mf/mg, ≥20 mf/mg skin) and sex as covariates.

Descriptive statistics SmfD by sex

Time point	Treatment Group	Sex	N	AM (SD)	AM 95% CI	Median (1Q,3Q)	Min·, Max·	GM (95%CI)
BL	Moxidectin	Male	626	39·2 (29·5)	[36·9,41·5]	31·0 (19·0,49·0)	9·0,299·0	31·5 [29·9,33·2]
	Ivermectin	Male	315	43·7 (33·7)	[40·0,47·4]	33·0 (18·0,60·0)	10·0,238·0	34·0 [31·4,36·8]
	Moxidectin	Female	352	38·2 (32·3)	[34·8,41·6]	27·5 (16·5,50·0)	10·0,212·0	29·6 [27·5,31·9]
	Ivermectin	Female	179	36·7 (26·1)	[32·8,40·5]	29·0 (15·0,53·0)	10·0,159·0	29·2 [26·4,32·3]
M12	Moxidectin	Male	604	1·7 (3·5)	[1·4,1·9]	0·0 (0·0,1·0)	0·0,28·0	0·8 [0·7,0·9]
	Ivermectin	Male	306	11·1 (13·5)	[9·6,12·6]	6·0 (2·0,15·0)	0·0,77·0	5·7 [4·9,6·6]
	Moxidectin	Female	343	0·5 (1·5)	[0·3,0·6]	0·0 (0·0,0·0)	0·0,13·0	0·2 [0·1,0·3]
	Ivermectin	Female	174	8·1 (11·6)	[6·3,9·8]	3·0 (1·0,10·0)	0·0,65·0	3·9 [3·2,4·8]

Descriptive statistics SmfD by baseline level of infection

Time point	Treatment Group	LoI	N	AM (SD)	AM 95% CI	Median (1Q,3Q)	Min·, Max·	GM (95%CI)
BL	Moxidectin	<20 mf/mg	281	13·9 (2·9)	[13·6,14·3]	13·0 (11·0,16·0)	9·0,19·0	13·7 [13·4,14·0]
	Ivermectin	<20 mf/mg	150	14·1 (2·9)	[13·6,14·5]	14·0 (11·0,17·0)	10·0,19·0	13·8 [13·3,14·2]
	Moxidectin	≥20 mf/mg	697	48·9 (30·9)	[46·6,51·2]	40·0 (28·0,60·0)	20·0,299·0	42·4 [40·8,44·1]
	Ivermectin	≥20 mf/mg	344	53·0 (30·7)	[49·7,56·2]	46·0 (30·0,66·5)	20·0,238·0	46·3 [43·8,48·9]
M12	Moxidectin	<20 mf/mg	270	0·4 (1·0)	[0·2,0·5]	0·0 (0·0,0·0)	0·0,8·0	0·2 [0·1,0·3]
	Ivermectin	<20 mf/mg	148	3·4 (6·0)	[2·5,4·4]	2·0 (1·0,4·0)	0·0,58·0	1·9 [1·6,2·4]
	Moxidectin	≥20 mf/mg	677	1·6 (3·4)	[1·3,1·8]	0·0 (0·0,1·0)	0·0,28·0	0·7 [0·6,0·8]
	Ivermectin	≥20 mf/mg	332	12·9 (14·1)	[11·4,14·4]	8·0 (3·0,18·0)	0·0,77·0	7·3 [6·4,8·3]

Mixed-effects model output

Time point	Treatment Group	N	Adjusted AM [95% CI]	Difference adjusted AM [95% CI]	AGM [95% CI]	Difference of AGM [95% CI]	Difference AGM in % AGM IVM	p-value [d]
M12	Moxidectin	947	0·5 [0·3,0·7]	1·2 [1·1,1·3]	0·6 [0·3,1·0]	3·9 [3·2,4·9]	86·4	<0·0001
	Ivermectin	480	1·7 [1·5,1·9]		4·5 [3·5,5·9]			

[d] p-value obtained from mixed-effects model, with baseline SmfD, treatment, sex, LoI, treatment*sex and treatment*LoI interactions as fixed effects and site as random effect.
Test of interactions: Treatment group * Sex: p-value= 0·0993, Treatment group * LoI at baseline: p <0·0001

Mixed-effects model output by sex and baseline level of infection (LoI)

Treatment	Results by	Adjusted AM [95% CI]	Difference of Adjusted AM [95% CI]	AGM [95% CI]	Difference of AGM [95% CI]	Difference between AGM in % AGM IVM	p-value[ao]
	**Sex**						
Moxidectin	FEMALE	0·3 [0·1;0·5]		0·4 [0·1;0·7]			
Ivermectin	FEMALE	1·6 [1·4;1·8]	1·3 [1·2;1·4]	4·0 [3·0;5·4]	3·7 [2·9;4·7]	90·8	<0·0001
Moxidectin	MALE	0·7 [0·4;0·9]		0·9 [0·5;1·4]			
Ivermectin	MALE	1·8 [1·6;2·0]	1·2 [1·0;1·3]	5·1 [3·9;6·6]	4·2 [3·3;5·2]	82·0	<0·0001
	**LoI**						
Moxidectin	< 20 mf/mg	0·6 [0·4;0·9]		0·9 [0·5;1·4]			
Ivermectin	< 20 mf/mg	1·5 [1·3;1·8]	0·9 [0·8;1·1]	3·7 [2·7;5·0]	2·8 [2·2;3·6]	75·6	<0·0001
Moxidectin	≥ 20 mf/mg	0·3 [0·1;0·5]		0·4 [0·1;0·7]			
Ivermectin	≥ 20 mf/mg	1·9 [1·7;2·1]	1·6 [1·5;1·7]	5·5 [4·2;7·1]	5·2 [4·1;6·4]	93·1	<0·0001

[a] p-value obtained from least square means pairwise comparisons based on the mixed-effect model.
Abbreviations: AM Arithmetic mean, BL pre-treatment/baseline, AGM adjusted Geometric Means, GM geometric mean, M12 Month 12, SD standard deviation

2.2.2 Protocol-planned non-parametric analysis of primary efficacy endpoint (SmfD 12 months post-treatment)

P value of non parametric model for treatment difference: p<0.0001. Interaction treatment * Sex: p=0.4250, Interaction treatment * level of infection at baseline: p <0.0001.

2.2.3 Sensitivity analysis of primary efficacy endpoint in response to statistical peer review: Fixed effects model

Table S 10: Primary efficacy endpoint: Fixed effects model sensitivity analysis output

Model output

Time point	Treatment Group	N	Adjusted AM [95% CI]	Difference adjusted AM [95% CI]	AGM [95% CI]	Difference of AGM [95% CI]	Difference AGM in % AGM IVM	p-value[d]
M12	Moxidectin	947	0·5 [0·4;0·5]	1·2 [1·1;1·3]	0·6 [0·3;0·7]	3·9 [3·6;4·3]	86·4	<0·0001
	Ivermectin	480	1·7 [1·6;1·8]		4·5 [4·1;5·0]			

[d] p-value obtained from fixed-effects model, with baseline SmfD, treatment, sex, LoI, treatment*sex and treatment*LoI interactions and site as fixed effects.
Test of interactions: Treatment group * Sex: p = 0·1001, Treatment group * LoI at baseline: p <0·0001

Fixed effects model output by sex and baseline level of infection (LoI)

Treatment	Results by	Adjusted AM [95% CI]	Difference adjusted AM [95% CI]	AGM [95% CI]	Difference of AGM [95% CI]	Difference AGM in % AGM IVM	p-value[ao]
	**Sex**						
Moxidectin	FEMALE	0·3 [0·2;0·4]		0·4 [0·3;0·5]			
Ivermectin	FEMALE	1·6 [1·5;1·7]	1·3 [1·2;1·4]	4·0 [3·5;4·7]	3·7 [3·2;4·2]	90·8	<0·0001
Moxidectin	MALE	0·7 [0·6;0·7]		0·9 [0·8;1·1]			
Ivermectin	MALE	1·8 [1·7;1·9]	1·2 [1·0;1·3]	5·1 [4·6;5·7]	4·2 [3·8;4·6]	82·0	<0·0001
	**LoI**						
Moxidectin	< 20 mf/mg	0·6 [0·5;0·8]		0·9 [0·7;1·1]			
Ivermectin	< 20 mf/mg	1·5 [1·4;1·7]	0·9 [0·8;1·1]	3·7 [3·1;4·4]	2·8 [2·4;3·3]	75·6	<0·0001
Moxidectin	≥ 20 mf/mg	0·3 [0·3;0·4]		0·4 [0·3;0·5]			
Ivermectin	≥ 20 mf/mg	1·9 [1·8;2·0]	1·6 [1·5;1·7]	5·5 [5·0;6·2]	5·2 [4·7;5·7]	93·1	<0·0001

a p-value obtained from least square means pairwise comparisons based on the fixed-effect model.
Abbreviations: AM Arithmetic mean, AGM adjusted Geometric Means, IVM ivermectin

2.2.4 Sensitivity analysis in response to statistical peer review: Descriptive statistics and linear mixed model of percentage SmfD change from baseline at month 12

Table S 11: Descriptive statistics and linear mixed model analysis output for percentage SmfD change from baseline at month 12

Descriptive statistics for SmfD change from baseline to 12 months post-treatment in percent of baseline SmfD

Time point	Treatment Group	N	AM (SD)	AM 95% CI	Median (1Q,3Q)	Min., Max.
M12	Moxidectin	947	96·8 (7·4)	[96·3;97·3]	99·7 (97·4,100·0)	41·5,100·0
	Ivermectin	480	76·5 (28·9)	[73·9;79·1]	84·7 (68·1,94·0)	-276,100·0

Model output

Time point	Treatment Group	N	Adjusted AM [95% CI]	Difference adjusted AM [95% CI]*	Difference Adj AM % Adj AM IVM	p-value[a]
M12	Moxidectin	947	96·8 [93·1;100·5]			
	Ivermectin	480	76·6 [72·7;80·5]	20·2 [18·1;22·4]	26·4	<0·0001

Test of interactions: Treatment group * Sex: p = 0·2724, Treatment group * LoI at baseline: p = 0·4981

Model output by sex and baseline level of infection (LoI)

Treatment	Results by	Adjusted AM [95% CI]	Difference of Adjusted AM [95% CI]*	p-value[ao]
	**Sex**			
Moxidectin	FEMALE	98·2 [94·2;102·2]		
Ivermectin	FEMALE	79·1 [74·7;83·5]	19·1 [15·9;22·4]	<0·0001
Moxidectin	MALE	95·4 [91·6;99·2]		
Ivermectin	MALE	74·0 [70·0;78·1]	21·4 [18·8;23·9]·	<0·0001
	**LoI**			
Moxidectin	< 20 mf/mg	96·7 [92·3;101·1]		
Ivermectin	< 20 mf/mg	75·7 [70·9;80·5]	21·0 [17·4;24·5]	<0·0001
Moxidectin	≥ 20 mf/mg	96·9 [93·1;100.7]		
Ivermectin	≥ 20 mf/mg	77·4 [73·3;81·4]	19.5 [17·1;21·9]	<0·0001

[a] p-value obtained from with % change from baseline at month 12 as outcome and with baseline SmfD, treatment, sex, LoI, treatment*sex and treatment*LoI interactions as fixed effects and site as random effect
*Difference in adjusted AM calculated as Adjusted AM Moxidectin - Adjusted AM Ivermectin. Note: for all other models the difference in adjusted AM and GM was calculated as Adjusted AM Ivermectin - Adjusted AM Moxidectin.
Abbreviations: AM Arithmetic mean.

2.2.5 Secondary efficacy endpoints

Table S 12: Skin microfilariae density 1, 6, 12 and 18 months after treatment: Descriptive statistics and mixed effects model output

Descriptive statistics

Time point	Treatment	N	Raw AM (SD)	Raw 95% CI	Raw Median (1Q,3Q)	Raw Min., Max.	Geometric Mean [95% CI]
Baseline	Moxidectin	978	38·8 (30·5)	[36·9, 40·8]	31·0 (18·0, 49·0)	9·0, 299·0	30·8 [29·5,32·1]
	Ivermectin	494	41·2 (31·3)	[38·4, 43·9]	32·0 (17·0, 56·0)	10·0, 238·0	32·2 [30·2,34·3]
Month 1	Moxidectin	973	0·1 (0·5)	[0·0, 0·1]	0·0 (0·0, 0·0)	0·0, 8·0	0·0 [0·0,0·0]
	Ivermectin	492	2·3 (7·2)	[1·7, 2·9]	0·0 (0·0, 1·0)	0·0, 54·0	0·7 [0·6,0·8]
Month 6	Moxidectin	962	0·0 (0·1)	[0·0, 0·0]	0·0 (0·0, 0·0)	0·0, 2·0	0·0 [0·0,0·0]
	Ivermectin	491	3·6 (6·2)	[3·1, 4·2]	1·0 (0·0, 4·0)	0·0, 43·0	1·8 [1·5,2·0]
Month 12	Moxidectin	947	1·2 (3·0)	[1·0, 1·4]	0·0 (0·0, 1·0)	0·0, 28·0	0·5 [0·5,0·6]
	Ivermectin	480	10·0 (12·9)	[8·8, 11·1]	5·0 (1·0, 13·0)	0·0, 77·0	5·0 [4·4,5·7]
Month 18	Moxidectin	764	4·3 (8·7)	[3·6, 4·9]	1·0 (0·0, 4·0)	0·0, 71·0	1·6 [1·4,1·8]
	Ivermectin	386	15·3 (18·3)	[13·5, 17·2]	8·0 (2·0, 21·0)	0·0, 101·0	7·7 [6·7,8·8]

Mixed effects model output by time post treatment

Treatment Group	Time post-treatment	Adjusted AM [95% CI]	Difference of Adjusted AM [95% CI]	AGM [95% CI]	Difference of AGM [95% CI]	Difference between AGM in % AGM IVM	p-value [a]
Moxidectin	Month 1	0·1 [-0·1;0·2]	0·4 [0·3;0·5]	0·1 [-0·1;0·3]	0·6 [0·5;0·7]	86·2	<0·0001
Ivermectin	Month 1	0·5 [0·3;0·7]		0·7 [0·4;0·9]			
Moxidectin	Month 6	0·1 [-0·1;0·2]	0·9 [0·9;1·0]	0·1 [-0·1;0·2]	1·6 [1·4;1·9]	96·8	<0·0001
Ivermectin	Month 6	1·0 [0·8;1·1]		1·7 [1·3;2·2]			
Moxidectin	Month 12	0·5 [0·3;0·6]	1·3 [1·2;1·3]	0·6 [0·4;0·9]·	4·1 [3·5;4·8}	86·6	<0·0001
Ivermectin	Month 12	1·7 [1·6;1·9]		4·7 [3·9;5·7]			
Moxidectin	Month 18	1·0 [0·9;1·2]	1·1 [1·0;1·2]	1·8 [1·4;2·3]	5·6 [4·7;6·6]	75·6	<0·0001
Ivermectin	Month 18	2·1 [2·0;2·3]		7·4 [6·1;8·9]			

Model output by level of infection at baseline (LoI)

Treatment Group	LoI	Adjusted AM [95% CI]	Difference of Adjusted AM [95% CI]	AGM [95% CI]	Difference of AGM [95% CI]	Difference between AGM in % AGM IVM	p-value [a]
Moxidectin	< 20 mf/mg	0·5 [0·4;0·7]	0·7 [0·6;0·8]	0·7 [0·4;1·0]	1·8 [1·5;2·2]	72·0	<0·0001
Ivermectin	< 20 mf/mg	1·3 [1·1;1·4]		2·5 [2·0;3·2]			
Moxidectin	≥ 20 mf/mg	0·3 [0·1;0·4]	1·1 [1·1;1·2]	0·3 [0·1;0·6]	2·8 [2·4;3·3]	89·2	<0·0001
Ivermectin	≥ 20 mf/mg	1·4 [1·3;1·6]		3·1 [2·5;3·8]			

Tests of Interaction: Treatment * sex: p-value=0·1652, Treatment * LoI p-value <0·0001, Treatment * Time point p-value <0·0001, obtained from a mixed-effects model with repeated measures with baseline SmfD, sex, LoI, treatment, time, treatment*sex, treatment*LoI and treatment*time interaction as fixed effects, time as repeated effect for the 4 SmfD measurements with subject as classification level.
[a] p-value obtained from least square means two-way comparisons based on the mixed-effects model.
Abbreviations: AM Arithmetic mean, AGM Adjusted Geometric Mean, GM Geometric mean,

Table S 13: Percentage SmfD change from baseline of skin microfilariae density 1, 6, 12 and 18 months after treatment: Descriptive statistics

Time point	Treatment Group	N	AM (SD)	AM 95% CI	Median (1Q,3Q)	Min., Max.
Month 1	Moxidectin	973	99·8 (0·6)	[99·8, 99·9]	100·0 (100·0, 100·0)	91·9, 100·0
	Ivermectin	492	93·9 (16·2)	[92·5, 95·4]	99·6 (96·6, 100·0)	-49·6, 100·0
Month 6	Moxidectin	962	99·9 (0·4)	[99·9, 100·0]	100·0 (100·0, 100·0)	91·5, 100·0
	Ivermectin	491	90·9 (15·0)	[89·6, 92·3]	95·8 (89·5, 98·8)	-59·3, 100·0
Month 12	Moxidectin	947	96·8 (7·4)	[96·3, 97·3]	99·7 (97·4, 100·0)	41·5, 100·0
	Ivermectin	480	76·5 (28·9)	[73·9, 79·1]	84·7 (68·1, 94·0)	-276, 100·0
Month 18	Moxidectin	764	89·1 (19·9)	[87·7, 90·5]	96·9 (87·0, 100·0)	-78·2, 100·0
	Ivermectin	386	64·0 (35·3)	[60·5, 67·6]	73·5 (49·5, 89·5)	-130, 100·0

AM Arithmetic mean, SD Standard deviation, 95% CI 95% Confidence interval,

Table S 14: Proportion (%) of participants with undetectable levels of skin microfilariae 1, 6, 12 and 18 months after treatment: Descriptive statistics and adjusted odds ratios

Time point	Treatment Group	Participants with undetectable microfilariae / participants with data	% with undetectable skin microfilariae	Odds Ratio [95% CI][1]	Odds Ratio [95% CI][2]	p-value[3]
Month 1	Moxidectin	813/973	83·6%	0·1287 [0·0995; 0·1667]		<0·0001
	Ivermectin	208/492	42·3%		7·77 [6·00; 10·05]	
Month 6	Moxidectin	881/962	91·6%	0·0070 [0·0045; 0·0109]		<0·0001
	Ivermectin	54/491	11·0%		141·9 [91·36; 220·4]	
Month 12	Moxidectin	440/947	46·5%	0·0424 [0·0268; 0·0670]		<0·0001
	Ivermectin	24/480	5·0%		23·59[14·92; 37·30]	
Month 18	Moxidectin	215/764	28·1%	0·0926 [0·0539; 0·1589]		<0·0001
	Ivermectin	16/386	4·1%		10·80 [6·29; 18·55]	

[1] Odds for undetectable SmfD after ivermectin treatment relative to odds for undetectable SmfD after moxidectin treatment obtained from the mixed effects logistic regression, see below.

[2] Odds for undetectable SmfD after moxidectin treatment relative to odds for undetectable SmfD after ivermectin treatment obtained from the mixed effects logistic regression, see below.

[3] p-values from mixed effects logistic model where undetectable skin microfilariae level (yes/no) is the outcome with treatment, sex and LoI as fixed effect and study site as random effect for each follow-up time point (1, 6, 12, 18 months).

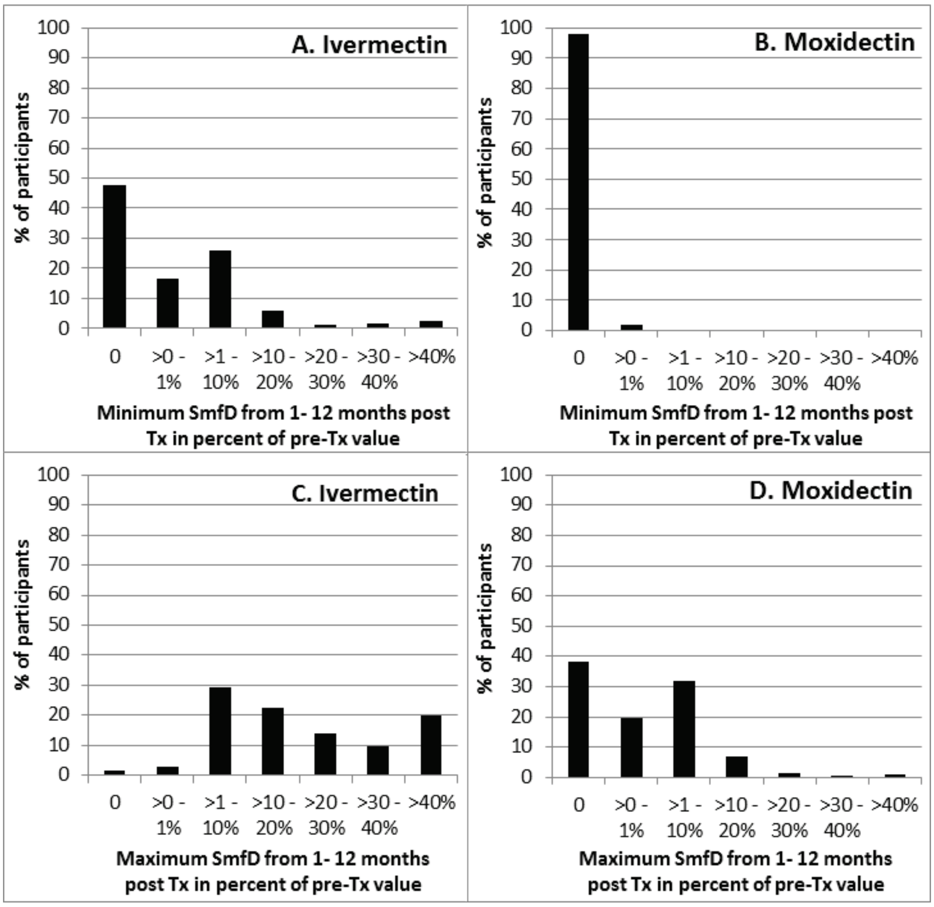

Figure S 3 Percentage of participants with 1, 6, and 12 months follow up data treated with ivermectin (A, C, n=478) and moxidectin (B, D, n=938) by minimum (A, B) and maximum (C, D) skin microfilariae densities between 1 and 12 months in percent of pre-treatment skin microfilariae density

Table S 15: Microfilariae levels in the anterior chamber 12 months post treatment among participants with > 10 mf across both anterior chambers at baseline and Month 12 data for both eyes: Descriptive statistics

Time point	Treatment Group	Number of participants	Raw Mean (SD)	Raw 95% CI	Raw Median (1Q, 3Q)	Raw Min, Max	Raw Geometric Mean [95% CI]
Baseline	Moxidectin	131	26·4 (19·9)	[22·9,29·8]	21·0 (14·0,28·0)	11·0,102·0	22·2 [20·1,24·4]
	Ivermectin	74	26·4 (18·5)	[22·1,30·7]	18·0 (14·0,33·0)	11·0,92·0	22·1 [19·4,25·3]
Day 3 or 4	Moxidectin	131	20·3 (19·3)	[17·0,23·7]	16·0 (9·0,24·0)	0·0,100·0	13·5 [11·2,16·2]
	Ivermectin	74	26·0 (22·8)	[20·7,31·3]	17·5 (12·0,30·0)	2·0,120·0	19·6 [16·4,23·4]
Month 1	Moxidectin	130	4·7 (10·9)	[2·8,6·6]	0·0 (0·0,4·0)	0·0,51·0	1·1 [0·7,1·6]
	Ivermectin	74	8·5 (17·6)	[4·4,12·5]	0·0 (0·0,8·0)	0·0,100·0	1·9 [1·1,3·0]
Month 6	Moxidectin	130	0·5 (2·4)	[0·1,0·9]	0·0 (0·0,0·0)	0·0,20·0	0·2 [0·1,0·3]
	Ivermectin	74	0·9 (5·8)	[-0·4,2·3]	0·0 (0·0,0·0)	0·0,49·0	0·1 [0·0,0·3]
Month 12	Moxidectin	131	0·3 (2·2)	[-0·1,0·7]	0·0 (0·0,0·0)	0·0,22·0	0·1 [0·0,0·1]
	Ivermectin	74	1·2 (6·4)	[-0·3,2·7]	0·0 (0·0,0·0)	0·0,54·0	0·2 [0·1,0·4]
Month 18	Moxidectin	115	0·1 (0·6)	[-0·0,0·2]	0·0 (0·0,0·0)	0·0,6·0	0·0 [-0·0,0·1]
	Ivermectin	61	1·7 (10·1)	[-0·9,4·3]	0·0 (0·0,0·0)	0·0,77·0	0·2 [0·0,0·5]

Table S 16: Reduction (%) from baseline of microfilariae levels in the anterior chamber 12 months post treatment among participants with > 10 mf across both anterior chambers at baseline and Month 12 data for both eyes): Descriptive statistics and model output

Time point	Treatment Group	N	Reduction (%) Mean(SD)	Reduction (%) [95% CI]	Reduction (%) Median(1Q, 3Q)	Reduction (%) Min, Max	Adjusted Mean Change from Baseline [95% CI]	Diff Adjusted Mean Change [95% CI]	p-Value
Month 12	Moxidectin	131	-99.3 (4.4)	[-100,-98.6]	-100 (-100,-100)	-100,-57.1	-98·5 [-101;-95.7]	-1·9 [-4.4;0.5]	0·1259
	Ivermectin	74	-97.3 (11.9)	[-100,-94.5]	-100 (-100,-100)	-100,-3.6	-96·5 [-99.5;-93.6]		

p-value obtained from mixed effect model model with sex, LoI, treatment, treatment*sex and treatment*LoI interactions as fixed effects and site as random effect.
Test of interaction: Treatment group * Sex: p-value= 0·0708, Treatment group * LoI: p-value= 0·2777

2.3 Safety data

2.3.1 Serious Adverse Events

The table below shows the number of participants who experienced an SAE within 6 months of treatment. The total number of SAEs was 39 in the moxidectin group and 18 in the ivermectin group.

Table S 17: Serious adverse events (SAEs) during the first 6 months post treatment (MedDRA coding)

MedDRA system organ class	MedDRA preferred term	Moxidectin (N=978)		Ivermectin (N=494)	
		n	(%)	n	(%)
Infections and infestations		23	2·4	13	2·6
	Abdominal abscess	0		1	0·2
	Abscess limb	1	0·1	0	
	Cellulitis	1	0·1	0	
	Fungal skin infection	1	0·1	0	
	Gastroenteritis	2	0·2	0	
	Malaria	15	1·5	9	1·8
	Peritonitis	1	0·1	0	
	Pneumonia	1	0·1	1	0·2
	Respiratory tract infection	0		2	0·4
	Sepsis	0		1	0·2
	Shigella infection	1	0·1	0	
Gastrointestinal disorders		7	0·7	5	1
	Abdominal pain	1	0·1	0	
	Abdominal pain lower	1	0·1	0	
	Abdominal pain upper	0		1	0·2
	Diarrhoea	1	0·1	3	0·6
	Enteritis	2	0·2	0	
	Gastritis	2	0·2	1	0·2
	Haematemesis	1	0·1	0	
Injury, poisoning and procedural complications		5	0·5	1	0·2
	Alcohol poisoning	1	0·1	0	
	Clavicle fracture	1	0·1	0	
	Contusion	0		1	0·2
	Head injury	1	0·1	0	
	Limb injury	1	0·1	0	
	Snake bite	1	0·1	0	
	Splenic rupture	1	0·1	0	

MedDRA system organ class	MedDRA preferred term	Moxidectin (N=978)		Ivermectin (N=494)	
Nervous system disorders		3	0·3	1	0·2
	Diabetic ketoacidotic hyperglycaemic coma	0		1	0·2
	Hemiplegia	1	0·1	0	
	Loss of consciousness	2	0·2	0	
	Meningism	0		1	0·2
	Cardiac disorders	2	0·2	0	
	Cardiac arrest	1	0·1	0	
	Cardiac failure congestive	1	0·1	0	
General disorders & administration site conditions		0		2	0·4
	Chills	0		1	0·2
	Influenza like illness	0		1	0·2
Respiratory, thoracic and mediastinal disorders		1	0·1	1	0·2
	Asthma	1	0·1	0	
	Cough	0		1	0·2
Eye disorders		1	0·1	0	
	Macular hole	1	0·1	0	
Hepatobiliary disorders		1	0·1	0	
	Hepatitis chronic active	1	0·1	0	
Metabolism and nutrition disorders		1	0·1	0	
	Dehydration	1	0·1	0	
Musculoskeletal and connective tissue disorders		1	0·1	0	
	Rheumatic disorder	1	0·1	0	
Reproductive system and breast disorders		1	0·1	0	
	Dysmenorrhoea	1	0·1	0	
Skin and subcutaneous tissue disorders		1	0·1	0	
	Skin ulcer	1	0·1	0	

2.3.2 Adverse events reported as resulting in Death (MedDRA coding)

Among moxidectin treated participants 2/978 (0·2%) and among ivermectin treated participants 2/494 (0·4%) died within 6 months of treatment. None of the AEs resulting in death (Table below) were assessed as related to study drug or Mazzotti reactions by the investigator.

Table S 18: Narratives generated from SAE reports for AEs with outcome death

The past medical history of this 63 year old woman showed abdominal pain upper (since 23-Dec-2010) and peptic ulcer.

She received **ivermectin** on 8 January 2010.

3 days before hospitalisation on 21 Mar 2010, she was reported to have difficulty in walking, fever, headache and pain in the lower limbs. On 21-Mar-2010 the patient was diagnosed with malaria and hospitalized. She was treated with quinine and paracetamol. On 22-Mar-2010, she developed meningeal syndrome (meningeal disorder) with symptoms of vomiting, neck stiffness, disorientation, psychomotor agitation and coma Grade II. She was treated with chloramphenicol. Thiamine was used as adjuvant treatment. On 23-Mar-2010 additional treatment medications were introduced and included ceftriaxone and dexamethasone. On 24-Mar-2010 the patient was diagnosed with diabetic acidotic coma and treatment with hydergine, tribexfort, bicarbonate and insulin were added. Thiamine was discontinued. On 25-Mar-2010 oxygen therapy was added and the patient remained in a coma.

Test results:

22-Mar-2010: white blood cell count (3.45 x10^3/L); neutrophil percentage (60 %); lymphocyte percentage (40 %); and haemoglobin (7.6 g/dL).

24-Mar-2010: blood glucose (18.88 mmol/L); blood pH (6, normal range: 7.38 - 7.42); and blood glucose (9.055 mmol/L).

25-Mar-2010: body temperature (37.9º C); blood glucose (9.055 mmol/L); blood pressure (100/70 mmHg); heart rate (results: 106 per minute); and respiratory rate (results: 32 per minute).

26-Mar-2010: Blood pH (venous 8.0, normal range: 7.38 - 7.42); blood glucose (10.77mmol/L).

27-Mar-2010: Blood glucose (13.33 mmol/L)

On 27-Mar-2010 the patient died. The cause of death was reported as diabetic coma, coma acidotic, malaria and meningeal disorder.

The investigator considered there was not a reasonable possibility that the event was related to the study

medication.
The relevant medical history of this 65 year old woman included flank pain on exertion, right atrial dilatation, epigastric pain and peptic ulcer syndrome<br>She received **ivermectin** on 20 August 2010.<br>On 22-Oct-2010, she experienced abdominal pain and asthenia and self medicated with an unspecified herbal therapy.<br>On 28-Oct-2010, the patient's condition was worsening and she was hospitalized. She was presenting fever, dizziness and painful micturition. Laboratory tests showed a haemoglobin level of 8.6 g/dL and trophozoite of plasmodium in a blood smear.<br>29-Oct-2010 test results: Widal test - TO 1/20 TH 1/40 (normal range 0-0); haemoglobin (results: 8.6 g/dL); urine analysis (microscopy and gram coloration: Gram + cocci, 5-10 WBC/Field (normal range 0) 1-5 epithelial cells (normal range 0)); blood smear test (1 trophozoite/100 field (normal range 0/100 field)); and white blood cell count (1700/ mm^3).<br>Treatment was started on 28-Oct-2010 with quinine and paracetamol, to which ceftriaxone, gentamycin, metronidazole were added on 29-Oct-2010. Blood transfusion was also given on 30-Oct-2010.<br>On 31-Oct-2010, the patient died. The cause of death was reported as septicemia.<br>The investigator considered there was not a reasonable possibility that the event was related to the study medication.
The past medical history of this 70 year old man included bronchial asthma.<br>He received **moxidectin** on 3 April 2010.<br>He started productive cough with dyspnoea on 07-Aug-2010. Physical examination on 07-Aug-2010 found presence of crackles and rhonchi all over the lung associated with tachycardia, fever and conjunctival hyperemia. Acute asthmatic attack (asthma) was diagnosed.<br>On 09-Aug-2010, the patient was hospitalized in the reference hospital.<br>Test results from 09-Aug-2010: blood bilirubin (Urobilinogen 33 mmol/L (normal less than 16 mmol/L)); urine ketone body ((Cetonuria) 1.5 mmol/L (normal - nil)); lymphocyte count (0.44 $x10^9$/L); neutrophil count (11.06 $x10^9$/L).<br>Treatment received included: salbutamol spray, hydrocortisone and artropine started on 07-Aug-2010, and aminophylline and erythromycin started on 08-Aug-2010.<br>He died on 11-Aug-2010. The cause of death was reported as asthma.<br>The investigator considered there was not a reasonable possibility that the event was related to the study medication.
The reported past medical history of this 60 year old woman did not include urinary incontinence and existence of a vesico vaginal fistula. She gave birth to the last child approximately 5 years before her death.<br>She received **moxidectin** on 24 November 2009.<br>She underwent a vesico-vaginal fistular surgical repair on 19-Jan-2010. Bloody urine was observed following the procedure which continued until 21-Jan-2010. Abdominal distension was observed proceeded by difficulty breathing, gasping and finally death. The patient's blood pressure declined during this period. The patient received ampicillin IV (post operative) starting on 19-Jan-2010, and chloramphenicol IV and flagyl IV were started on 21-Jan-2010.<br>14-Jan-2010 test results: haematocrit (33 %) and haemoglobin (11 g/dL).<br>19-Jan-2010 test results: haemoglobin (9.6 g/dL); haematocrit (29 %); blood pressure (130/80 mmHg).<br>20-Jan-2010 test results: blood pressure 130/80 mmHg.<br>21-Jan-2010 test results blood pressure 90/50 mmHg and later in day 80/40 mm Hg.<br>The patient died on 21-Jan-2010. The cause of death was reported as peritonitis and cardiac arrest.<br>The investigator considered there was not a reasonable possibility that the event was related to the study medication.

2.3.3 Dependency of Mazzotti reactions on pre-treatment skin microfilariae density

The subsequent figures show the percentage of participants with Mazzotti reactions overall and by highest grade experienced across all participants and by pre-treatment SmfD in consideration of the known dependency of post-ivermectin MAZ on pre-treatment SmfD.

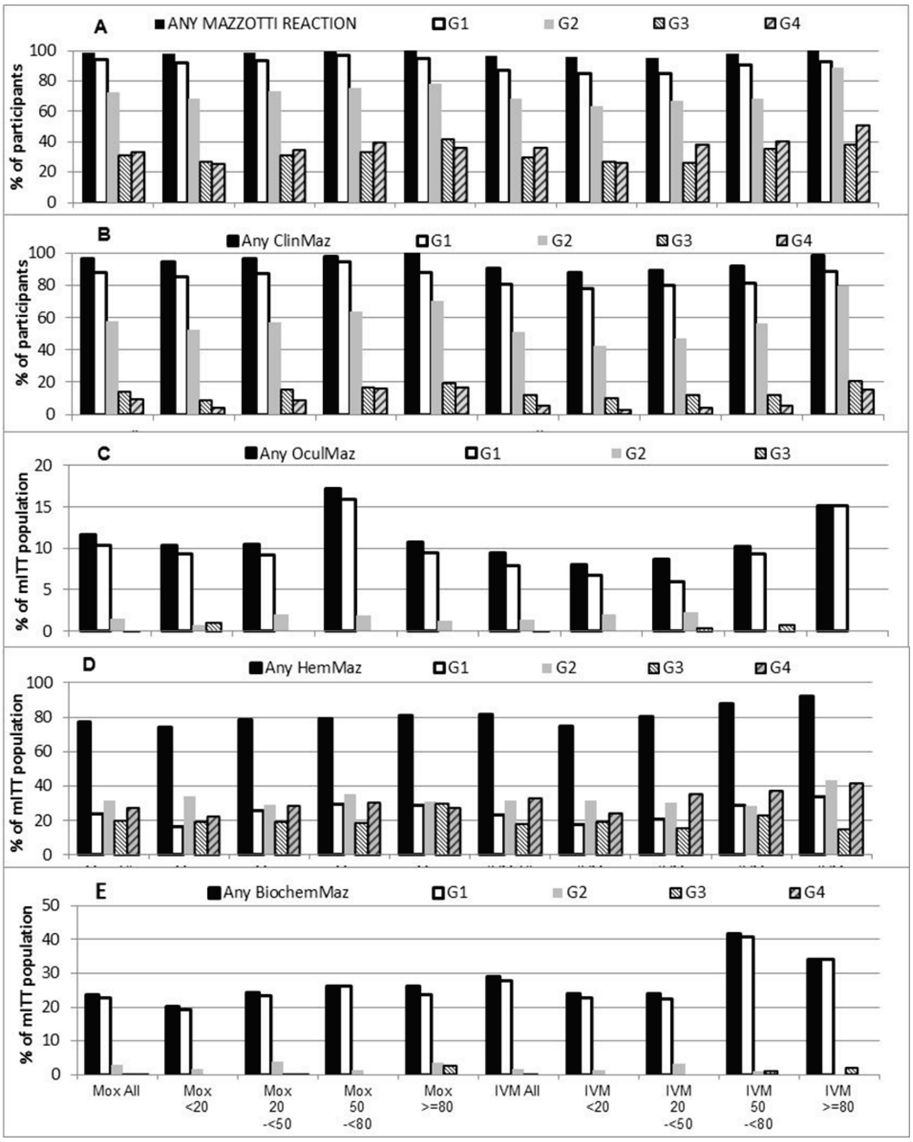

Figure S 4 Percentage of participants overall and by skin microfilariae density pre-treatment with at least one MAZ of any grade and by highest grade experienced. (A) Any type of MAZ, (B) any type of clinical MAZ, (C) any type of ocular MAZ, (D) any type of haematological MAZ, (E) any type of biochemical MAZ.

Legend: ClinMaz: Clinical Mazzotti reaction, OcularMaz: ocular Mazzotti reaction, HemMaz: haematological Mazzotti reaction, BiochemMaz: serum biochemical Mazzotti reactions, ANY: Mazzotti reaction of any grade; G1, G2, G3, G4: Highest experienced grade was grade 1, 2, 3, 4, respectively. Since an individual participant may have experienced more than one type of MAZ, the % of participants in individual MAZ cluster/group/grade categories is not necessarily identical to the % of participants at a higher level of MAZ cluster/group/grade category.

Mox: moxidectin, IVM: ivermectin. <20, 20-<50, 50-<80, >=80: pretreatment SmfD in mf/mg skin. Number of participants with pre-treatment skin microfilaria density of <20, 20 to <50, 50 to <80 and ≥80 was 281, 456, 157 and 84, respectively in the moxidectin and 150, 183, 108 and 53 in the ivermectin treatment arm.

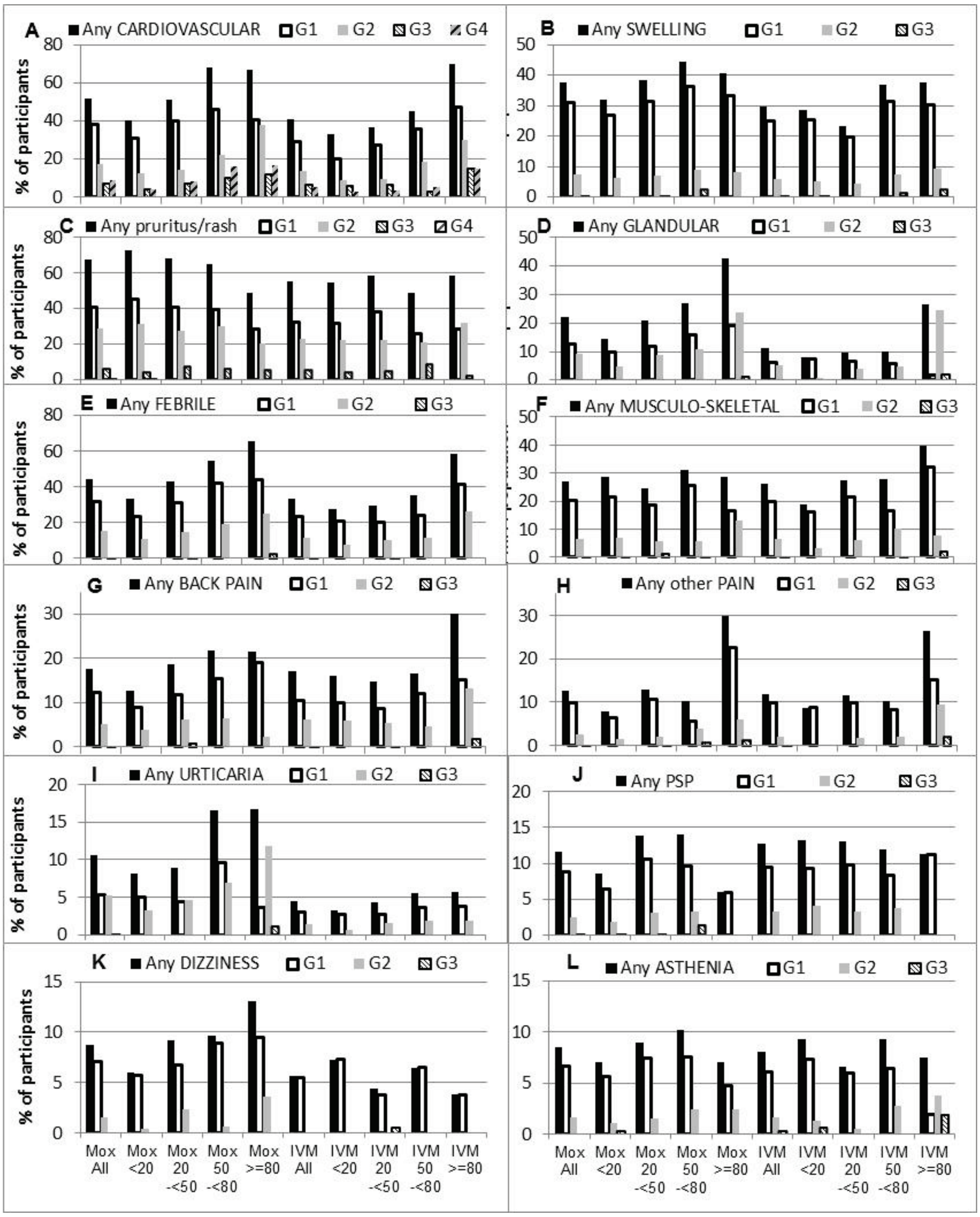

Figure S 5 Percentage of participants overall and by intensity of infection pre-treatment with at least one clinical MAZ of any grade and by highest grade experienced for the specified clinical MAZ group.

Legend: see Figure S4, PSP peripheral sensory phenomena.

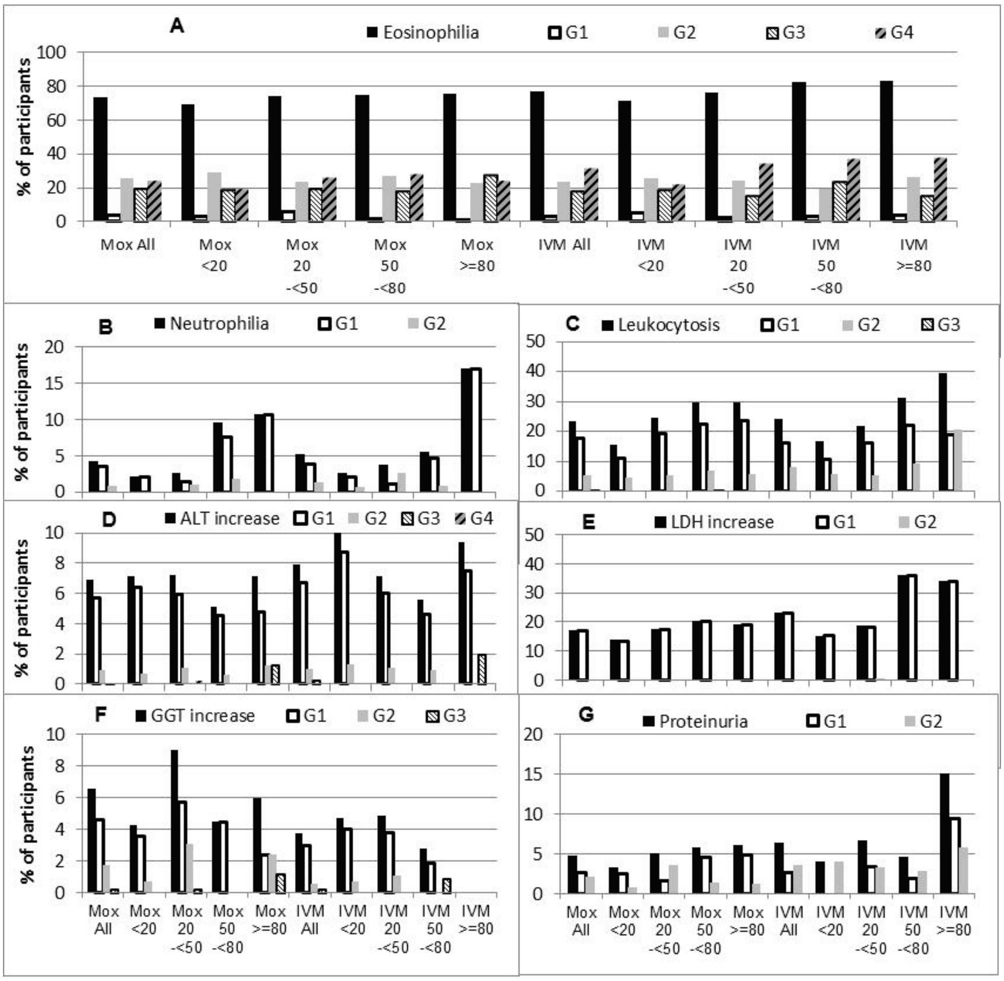

Figure S 6 Percentage of participants overall and by intensity of infection pre-treatment with the specified hematological or serum biochemical MAZ by highest grade experienced

Legend: see Figure S4

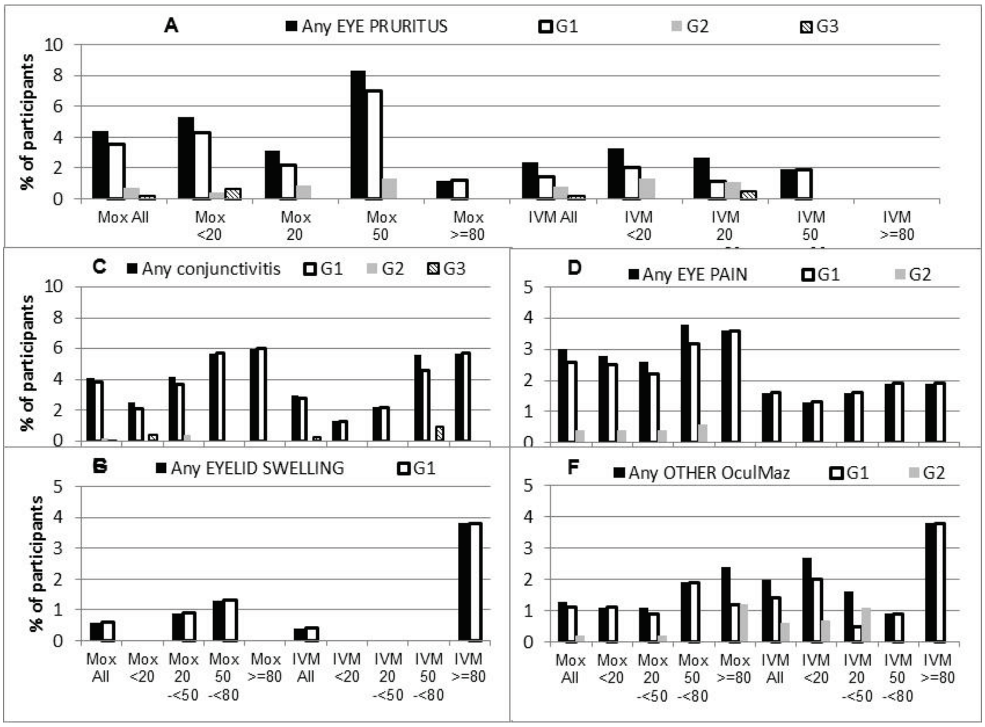

Figure S 7 Percentage of participants overall and by intensity of infection pre-treatment with the specified types of ocular MAZ of any grade and by highest grade experienced

Legend: see Figure S4, Any other ocular MAZ includes ocular discomfort, tearing/watery eyes, blurred vision, photophobia and visual acuity. The relatively high prevalence of other ocular MAZ among those with ≥80 mf/mg treated with ivermectin is due to tearing/watery eyes.

2.4 Exploratory endpoint: Effect on intestinal helminths

Intestinal helminths were only an exploratory endpoint. While the data for the primary and secondary endpoints were 100% source data verified, this was not the case for intestinal helminths.

Arithmetic mean based egg reduction rate is the WHO recommended method for quantifying drug efficacy[13] and provided together with cure rates in Table S16.

Table S 19: Cure rate and egg reduction rate across all sites for hookworm, roundworm and whipworm

	Moxidectin			Ivermectin		
**ERR[1]**	**EPG_{AM} pre-treatment[2]**	**EPG_{AM} at Month 1**	**ERR (%)**	**EPG_{AM} pre-treatment**	**EPG_{AM} at month 1**	**ERR (%)**
**Hookworm**	564	109	81	764	419	45
**Roundworm**	386	11	97	79	0	100
**Whipworm**	1409	7	99	1133	0	100
**Cure rates[3]**	**No infected pre-treatment[4]**	**No with undetectable infection Month 1**	**Cure rate (%)**	**Infected pre-treatment[4]**	**No with undetectable infection Month 1**	**Cure rate (%)**
**Hookworm**	472	222	47	245	69	28
**Roundworm**	34	33	97	7	7	100
**Whipworm**	11	10	91	5	5	100

[1] ERR = (EPG_{AM} pre-treatment – EPG_{AM} at Month 1 visit) / EPG_{AM} pre-treatment * 100
[2] EPG eggs/gram faeces, EPG_{AM} Arithmetic mean EPG.
[3] (Number of participants with epg >0 pre-treatment and epg = 0 at Month 1 follow up visit) / Number of participants with epg >0 pre-treatment * 100
[4] Number lower than those with infection detected pre-treatment since all participants who received mebendazole between screening and month 1 helminth evaluation are excluded from the analysis

Olliaro et al. suggested that the utility of two different individual data based methods for quantifying drug response be examined: (a) the distribution of proportions cured, with partial response (decrease in egg/g faeces) or no response (no change or increase in EPG) and (b) the centile distribution of individual egg reduction rates (ERRic).[14] The analysis of the data for hookworm, for which sufficient individual data were available is shown in Figure S 7.

Figure S 8 Individual data based analysis of response of hookworm infection to moxidectin and ivermectin

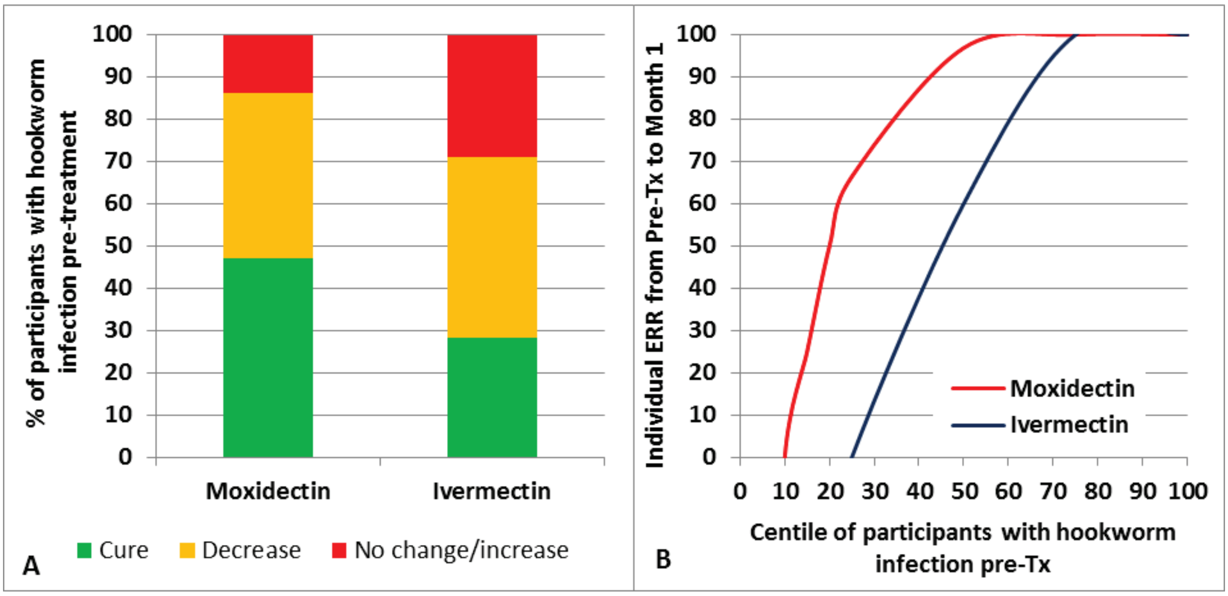

A. Percentage of participants with no change or increase in hookworm eggs/gram faeces (EPG), decrease in EPG or undetectable EPG (cure) 1 months post-treatment. B Centiles of participants by hookworm EPG reduction rate.

3 Classification of Response to ivermectin in previous studies

The table below provides an overview of criteria used in studies assessing adequate/expected/normal response vs. a lower level of response. Responses indicating a low sensitivity to the embryostatic effect of ivermectin are referred to frequently as suboptimal response (SOR)[15], responses indicating a low sensitivity to the 'microfilaricidal' effect of ivermectin have been referred to as SOMR[12].

In addition, the results of a meta-analysis of 26 clinical and field studies of skin microfilariae levels after a single dose of ivermectin are included (for an analyses of these data quantifying inter-subject variability in response as a basis for detecting suboptimal response to ivermectin, see Churcher et al.[16]).

Table S 20: Criteria in previous studies for classification of response to ivermectin treatment based on skin microfilariae levels

Time post-treatment	Evaluation unit<br>Response characterization	Skin mf density criteria	Quantitation of SmfD	Ivermectin treatment history<br>Reference
	Individual			None (first treatment)
8 days	Adequate response:	< 40% of pre-treatment	mf/4mg, 4 snips	Awadzi et al. [15]
90 days	Adequate response:	≤ 6% of pre-treatment		
365 days	Adequate response:	≤ 40% of pre-treatment		
	Community			≥ 6 rounds of CDTI
90 days	Expected response:	<6% of pre-treatment	mf/snip, 2 snips	Osei-Atweneboana et al.[17]
180 days	Normal response:	<20% of pre-treatment		
180 days	Poor response, alarming response:	>30 % of pre-treatment		
	Community			≥ 6 rounds of CDTI
364 days	Good response:	< pre-treatment	mf/snip, 2 snips	Osei-Atweneboana et al.[18]
364 days	Intermediate response:	≈ pre-treatment		
364 days	Poor response:	> pre-treatment		
	Meta-analysis of 26 clinical and field studies			None (first treatment)
1 week	Model predicted:	<6% of pre-treatment GM		Basanez et al. [19] (meta-analysis)
1-2 months	Model predicted:	1 % of pre-treatment GM		
2-10 months	Model predicted:	< 20% of pre-treatment GM		
12 months	Model predicted:	≈ 20% of pre-treatment GM		

CDTI: Annual community directed treatment with ivermectin, GM Geometric Mean

4 References

(1) Awadzi K, Dadzie KY, DeSole G., Remme J. Reactions to ivermectin treatment in onchocerciasis patients. Acta Leiden. 1990;59(1-2):193-9.

(2) Hero M, Bird AC, Awadzi K. Quantification of the ocular reactions to microfilaricides in the chemotherapy of onchocerciasis. Eye (Lond). 1992;6 (Pt 1):93-6.

(3) Awadzi K. The chemotherapy of onchocerciasis II. Quantitation of the clinical reaction to microfilaricides. Ann Trop Med Parasitol. 1980 Apr;74(2):189-97.

(4) Francis H, Awadzi K, Ottesen EA. The Mazzotti reaction following treatment of onchocerciasis with diethylcarbamazine: clinical severity as a function of infection intensity. Am J Trop Med Hyg. 1985 May;34(3):529-36.

(5) Baraka OZ, Mahmoud BM, Ali MM, Ali MH, el Sheikh EA, Homeida MM, et al. Ivermectin treatment in severe asymmetric reactive onchodermatitis (sowda) in Sudan. Trans R Soc Trop Med Hyg. 1995 May;89(3):312-5.

(6) Awadzi K, Opoku NO, Addy ET, Quartey BT. The chemotherapy of onchocerciasis. XIX: The clinical and laboratory tolerance of high dose ivermectin. Trop Med Parasitol. 1995 Jun;46(2):131-7.

(7) Bryceson AD, Warrell DA, Pope HM. Dangerous reactions to treatment of onchocerciasis with diethylcarbamazine. Br Med J. 1977 Mar 19;1(6063):742-4.

(8) Awadzi K, Gilles HM. Diethylcarbamazine in the treatment of patients with onchocerciasis. Br J Clin Pharmacol. 1992 Oct;34(4):281-8.

(9) Remme J, Baker RH, DeSole G, Dadzie KY, Walsh JF, Adams MA, et al. A community trial of ivermectin in the onchocerciasis focus of Asubende, Ghana. I. Effect on the microfilarial reservoir and the transmission of Onchocerca volvulus. Trop Med Parasitol. 1989 Sep;40(3):367-74.

(10) Albiez EJ, Büttner D, Duke BO. Diagnosis and extirpation of nodules in human onchocerciasis. Trop Med Parasitol. 1988 Dec;39 Suppl 4:331-46.

(11) Fischer P, Kipp W, Bamuhiga J, Binta-Kahwa J, Kiefer A, Buttner DW. Parasitological and clinical characterization of *Simulium neavei*-transmitted onchocerciasis in western Uganda. Trop Med Parasitol. 1993 Dec;44(4):311-21.

(12) Awadzi K, Opoku NO, Attah SK, Lazdins-Helds J, Kuesel AC. A Randomized, Single-Ascending-Dose, Ivermectin-Controlled, Double-Blind Study of Moxidectin in Onchocerca volvulus Infection. PLoS

Negl Trop Dis. 2014 Jun;8(6):e2953. doi: 10.1371/journal.pntd.0002953 [doi];PNTD-D-13-00311 [pii].

(13) World Health Organization. Assessing the efficacy of anthelminthic drugs against schistosomiasis and soil-transmitted helminthiases. Geneva: World Health Organization; 2013.

(14) Olliaro PL, Diawara A, Speich B, Keiser J, Halpenny C, Albonico M, et al. Comparing group means and individual responses to treatment of soil-transmitted nematodes with benzimidazole drugs: a pooled analysis of individual patient data. Annual Meeting ASTMH (https://www.astmh.org/ASTMH/media/Documents/ASTMH2014AbstractBookFINAL.pdf) Abstract 642, 194. 2014.

(15) Awadzi K, Boakye DA, Edwards G, Opoku NO, Attah SK, Osei-Atweneboana MY, et al. An investigation of persistent microfilaridermias despite multiple treatments with ivermectin, in two onchocerciasis-endemic foci in Ghana. Ann Trop Med Parasitol. 2004 Apr;98(3):231-49.

(16) Churcher TS, Pion SD, Osei-Atweneboana MY, Prichard RK, Awadzi K, Boussinesq M, et al. Identifying sub-optimal responses to ivermectin in the treatment of River Blindness. Proc Natl Acad Sci U S A. 2009 Sep 29;106(39):16716-21.

(17) Osei-Atweneboana MY, Eng JK, Boakye DA, Gyapong JO, Prichard RK. Prevalence and intensity of *Onchocerca volvulus* infection and efficacy of ivermectin in endemic communities in Ghana: a two-phase epidemiological study. Lancet. 2007 Jun 16;369(9578):2021-9.

(18) Osei-Atweneboana MY, Awadzi K, Attah SK, Boakye DA, Gyapong JO, Prichard RK. Phenotypic Evidence of Emerging Ivermectin Resistance in *Onchocerca volvulus*. PLoS Negl Trop Dis. 2011;5(3):e998.

(19) Basanez MG, Pion SD, Boakes E, Filipe JA, Churcher TS, Boussinesq M. Effect of single-dose ivermectin on Onchocerca volvulus: a systematic review and meta-analysis. Lancet Infect Dis. 2008 May;8(5):310-22. doi: S1473-3099(08)70099-9 [pii];10.1016/S1473-3099(08)70099-9 [doi].
